# Supplementary material for: Thermodynamically stable whilst kinetically labile coordination bonds lead to strong and tough self-healing polymers
Source: Nat Commun. 2019 Mar 11;10:1164. doi: 10.1038/s41467-019-09130-z (PMC6411951; doi:10.1038/s41467-019-09130-z)
Supplement: Supplementary file 1 — Supplementary Information [file 41467_2019_9130_MOESM1_ESM.pdf]

Supplementary Information for

Thermodynamically stable whilst kinetically labile coordination bonds lead to mechanically robust self-healing polymers

Lai *et al.*

Supplementary Note 1: Results of model complex study

According to the reference 35 in the main text, the definition of "alterdentate ligand" is that a species which offers to a metal ion more than one equivalent coordination site. In an "alterdentate ligand" there is, principally, always a re-arrangement possibility in which the metal is transferred from one site to another one. This can be either an inter- or intra- molecular process. The rearrangement reaction is kinetically controlled by the activation energy and entropy experienced by the metal on the reaction path. The free energy difference is zero by definition, if the coordination sites are equivalent.

In order to determine the specific coordination properties of the Zn(II) complexes with the 2,6-bis((imino)methyl)-4-chlorophenol alterdentate ligand, we prepared a model ligand 2,6-bis((propylimino)methyl)-4-chlorophenol (Pr-Hbimcp, Supplementary Figure 36b), and studied its complexation with Zn(II).

1.1 Different coordination structure at different metal-to-ligand molar ratio

We performed the experiments with the $ZnCl_2$ to Pr-Hbimcp molar ratio as 1:2 and 1:1, to evaluate the impact of metal-to-ligand molar ratio to the formed structure of complex. When the molar ratio was 1:2, the product was an amorphous solid, which could be due to the dynamic properties of the formed complex. According to the ESI mass spectra (ESI-MS), the cationic complex $[Zn(Pr\text{-}Hbimcp)_2]^{2+}$, in which the Zn(II) ions were coordinated to two oxygen and two nitrogen atoms with four equivalent conformations[35](Figure 1a), has the maximum abundance (except for the free ligand fragments generated due to electrospray ionization, Supplementary Figure 2a). When the molar ratio was changed to 1:1, the neutral complex $Zn_2(Pr\text{-}bimcp)_2Cl_2$ dominate gradually in the products (Supplementary Figure 2 and Figure 3).

1.2 Crystal structure of $Zn(Pr\text{-}Hbimcp)Cl_2$, $Zn_2(Pr\text{-}bimcp)_2Cl_2$ and $[Zn_2(Pr\text{-}bimcp)(Pr\text{-}Hbimcp)_2(CH_3O)](ClO_4)_2$

Crystal structure of complexes $Zn(Pr\text{-}Hbimcp)Cl_2$, $Zn_2(Pr\text{-}bimcp)_2Cl_2$ and $[Zn_2(Pr\text{-}bimcp)(Pr\text{-}Hbimcp)_2(CH_3O)](ClO_4)_2$ are shown in Supplementary Figure 3. Selected bond lengths and angles are listed in Supplementary Table 2, Table 3-5.

In the structure of $Zn(Pr\text{-}Hbimcp)_2Cl_2$, each Zn(II) atom are tetra-coordinated with one imine nitrogen and one phenol oxygen atoms and two chloride atoms. The Zn(II) centers adopt slightly distorted tetrahedron geometry. The Zn−N and Zn−O bond distances for complex are 2.009(3) and 1.969(3) Å, the Zn-Cl bond distances for complex lie in the range 2.2248(11)−2.2389(12) Å.

The structure of $Zn_2(Pr\text{-}bimcp)_2Cl_2$ shows that the complex is a diphenoxo-bridged $Zn(II)_2$ system derived from the two bisimino phenolate ligand $[L]^-$. The diphenoxo-bridged di-nuclear moiety is neutral in which the two metal ions are coordinated to two anionic ligands and two chlorides. Each of the two $N(imine)_2O(phenolate)_2$ compartments are coordinated to two metal ions. The Zn(II) centers are penta-coordinated and adopt slightly distorted square pyramidal geometry in which the two imine nitrogen and two phenoxo oxygen atoms form the basal plane. The slightly distorted square pyramidal environment in each case is evidenced by the discrimination parameter (τ) value, which fall in the range of 0.0003−0.1323. The Zn−N and Zn−O bond distances for complex lie in the range 2.057(6)−2.070(5) Å and 2.052(4)−2.061(4) Å, respectively.

The structure of $[Zn_2(Pr\text{-}bimcp)(Pr\text{-}Hbimcp)_2(CH_3O)](ClO_4)_2$ shows that two Zn(II) centers are coordinated to three Hbimcp ligands and one methoxy anion. Each Zn(II) centers are penta-coordinated and adopt slightly distorted square pyramidal geometry. One of the Hbimcp ligand was deprotonated and the deprotonated phenoxo oxygen atom functioned as a bridge to connect the two Zn(II) centers. The Zn−N and Zn−O bond distances for complex lie in the range 2.037(6)-2.094(6) Å and 1.970(4)- 2.072(4) Å, respectively.

1.3 Determination of average coordination number and association constants using model ligands

UV-Vis spectroscopy was used to determine the coordination number and association constants of the model ligand and Zn(II), and the model ligand used in measurements was Pr-Hbimcp.

In order to dissolve the Pr-Hbimcp, metal salt and the complex products, we chose a mixed solvent with the DCM and methanol ratio as 9:1. Firstly, 5.32 mg Pr-Hbimcp and 2.74 mg $ZnCl_2$ were dissolved in 10 mL mixed solvent to get solution with the constant concentration of 10^{-3} M, respectively. Then we mix the ligand solution and Zn(II) solution with the volume ratio of $V_r = V_L/(V_L+V_M)$ range for 0.0 to 1.0 and measured with UV-Vis spectra (Supplementary Figure 16a). V_L and V_M represent the volume of ligand solution and Zn(II) solution. In order to calculate the association constant, another set of measurements were carried by diluting the ligand and metal solution into 5×10^{-4} M (Supplementary Figure 16b). Supplementary Figure 16 shows the UV-Vis spectra of the mix solution with different volume ratio V_r. With the increase of V_r, a new absorption band of complex at 422 nm was appear and reach the maximum value when $V_r = 0.6$. When $V_r = 1$, it shows the ligand absorption spectra with the maximum absorption value at 353 nm and a weak absorption value at 451 nm. According to the lambert-beer's law, we know that the absorbance A and concentration c follow the Supplementary Equation 1 as:

$$A = \frac{k}{2.303} cd \quad (1)$$

Where k and d is a constant, A and c follow the linear relation.

The maximum absorption values of complex with different V_r are employed to investigate coordination numbers between ligand and metal ions. The average coordination number (n) is defined in Supplementary Equation 2 as:

$$n = V_L/V_M \quad (2)$$

Supplementary Figure 17a shows the average coordination numbers between ligand and Zn(II) was about 1.5:1, which means that the coordination number will increasing from 2:1 to 1:1 with the increasing of Zn(II) volume. Such result is consistent with the ESI-MS and crystal results.

It also shows that when V_L= 1, the absorption at 422 nm was not equal to zero, indicating the presence of free ligand. In order to get the pure complex absorption value,

the absorption values from 0.0 to 1.0 were calibrated by subtracting the residual absorption of ligand, as show in Supplementary Figure 17b.

In this model complex study, all the complexation reactions can be expressed in Supplementary Equation 3 as:

$$x\,\mathrm{M} + y\,\mathrm{L} = \mathrm{M}_x\mathrm{L}_y \quad (3)$$

where M and L represent the metal ion Zn(II) and the ligand Pr-Hbimcp. Thus, the association constant can be expressed in Supplementary Equation 4 as:

$$K_s = \frac{c_{M_xL_y}}{c_M^x c_L^y} \quad (4)$$

Where the C_M and C_L represent the concentration of free metal ion and ligand in the reaction solution.

Define the original solution concentration as *a* and *b*, and the complex concentration as *m* when the coordination reaction was equilibrium, then the Supplementary Equation 4 can be expressed in Supplementary Equation 5 as:

$$K_s = \frac{m}{(a-m)^x(b-m)^y} \quad (5)$$

For the same complex, the same absorption means the same concentration. So we selected the same absorption points from the different solution concentration curves (Supplementary Figure 17b), as (a_1, b_1) and (a_2, b_2), then we could get the Supplementary Equation 6 as follow:

$$K_s = \frac{m}{(a_1-m)^x(b_1-m)^y} = \frac{m}{(a_2-m)^x(b_2-m)^y} \quad (6)$$

With the specific value of x and y, Then we can get the value of m and K_s: When $x = 1$ and $y = 2$, corresponding to the complex [Zn(Pr-Hbimcp)$_2$]Cl$_2$, then $K_{s1\text{-}2} = 2.2 \times 10^{11}$; When $x = 2$ and $y = 2$, corresponding to the complex [Zn$_2$(Pr-bimcp)$_2$Cl$_2$], then $K_{s2\text{-}2} = 3.7 \times 10^{17}$.

The association constants of complexes formed between Pr-Hbimcp and Ni^{2+} were measured with the same method except the metal ion used the Ni^{2+} (Supplementary Figure 19). We got a $K_{s1\text{-}2(Ni)} = 5.4\times10^{11}$ for complex [Ni(Pr-Hbimcp)$_2$]Cl$_2$.

Supplementary Note 2: Details of general measurements

The rheological properties were studied by a DHR-2 Rheometer (TA Instruments). Frequency and temperature sweeps were measured on circular samples (20 mm diameter). Frequency sweeps from 0.01 to 100 Hz were performed at 0.1% strain under 25 ºC. Temperature sweeps were measured from -5 ºC to 120 ºC at the different frequency, which strain was automatically modulated at 0.3% ± 0.2% to keep the measured torque at a reasonable value. The auto-compression feature was set to 0.2 ± 0.15 N in order to make samples well contacted.

Uniaxial tensile measurements were performed on an Instron 3343 equipped with a 500 N load cell with a specific strain rate at 25 °C. The testing rectangular films had the effective gauge dimensions with the length of 5 mm, width of 3 mm and thickness of 0.8 ~ 1 mm. Rectangular tensile bars were cut from large original polymer films using a blade. Each result was the average from at least three samples.

During the **self-healing test**, the polymer film was cut into two pieces and then put together and healed for different durations. The strain-stress curves of the healed samples were collected by a similar process as the original ones. The mechanical healing efficiency, η, was calculated from the fracture strain ratio between the restored one and the original one. The Young's modules, maximal strengths, breaking strengths, breaking strains, and healing efficiencies were presented as the mean ± standard deviation according to data of ≥ 5 trials.

Stress-relaxation analysis (SRA) was measured on an Instron 3343 utilizing rectangular films (ca.1 mm (T) × 3 mm (W) × 5 mm (L)). The SRA experiments were measured in a strain control (25% - 500% strain) mode at 25 °C under a strain rate of 50 mm min^{-1}. After equilibrating at 25 °C for about 5 minutes, the strain was applied and stress decay was monitored.

Single-Molecule Force Spectroscopy Study. Single molecule force experiments on **Hbimcp-PDMS** and **Zn(Hbimcp)$_2$-PDMS** macromolecules were performed on a modified AFM. A toluene solution of 150 mg mL^{-1} was used for the experiment of

Hbimcp-PDMS. For the experiment of **Zn(Hbimcp)$_2$-PDMS**, the toluene solution (150 mg mL^{-1}) of **Hbimcp-PDMS** was diluted to a final concentration of 15 mg mL^{-1} with MeOH/Toluene (*v*/*v* = 1/10) solution of $ZnCl_2$ (5 mg mL^{-1}). In order to investigate the reversibility of **Zn(Hbimcp)$_2$-PDMS** under the unfolding and stretching process, we released the unfolded **Zn(Hbimcp)$_2$-PDMS** chain quickly to zero force. The **Zn(Hbimcp)$_2$-PDMS** chain was stretched again after 1 s to probe whether it could fold back to its original state.

X-ray Structure Determination. Single crystals of Zn(Pr-Hbimcp)Cl_2 and Zn_2(Pr-bimcp)$_2Cl_2$ suitable for X-ray diffraction analysis were isolated from the reaction solution between $ZnCl_2$ and Pr-Hbimcp ligand in 1:2 and 1:1 molar ratio, respectively. Single crystals of [Zn_2(Pr-bimcp)(Pr-Hbimcp)$_2$(CH_3O)] (ClO_4)$_2$ suitable for X-ray diffraction analysis were obtained by adding $AgClO_4$ into CH_2Cl_2 solution of Zn(Pr-Hbimcp)Cl_2. The crystal structures were determined at 296 K on a Bruker SMART CCD diffractometer using monochromated Mo Kα radiation (λ = 0.71073 Å). The cell parameters were retrieved using SMART software and refined using *SAINT* for all observed reflections. The data were collected using a narrow-frame method with scan width of 0.3° in ω and an exposure time of 5 s per frame. The redundant data sets were reduced using *SAINT* [S1] and corrected for Lorentz and polarization effects. The absorption corrections were applied using *SADABS* [S2]. The structure was solved and refined using *SHELXLTL*.[S3] Direct method yielded all non-hydrogen atoms, which were refined using anisotropic thermal parameters. All hydrogen atom positions were calculated geometrically and were set riding on their respective atoms. More details for the data collections and structure refinements are given in Supplementary Table 3. Selected bond lengths, structural parameters and bond angles for complexes [Zn_2(bimcp)$_2Cl_2$] are listed in Supplementary Table 4-6. CCDC files (1886156 for Zn(Pr-Hbimcp)Cl_2, 1817755 for Zn_2(Pr-Hbimcp)$_2Cl_2$ and 1886157 for [Zn_2(Pr-bimcp)(Pr-Hbimcp)$_2$(CH_3O)](ClO_4)$_2$ contain the supplementary crystallographic data for this paper.

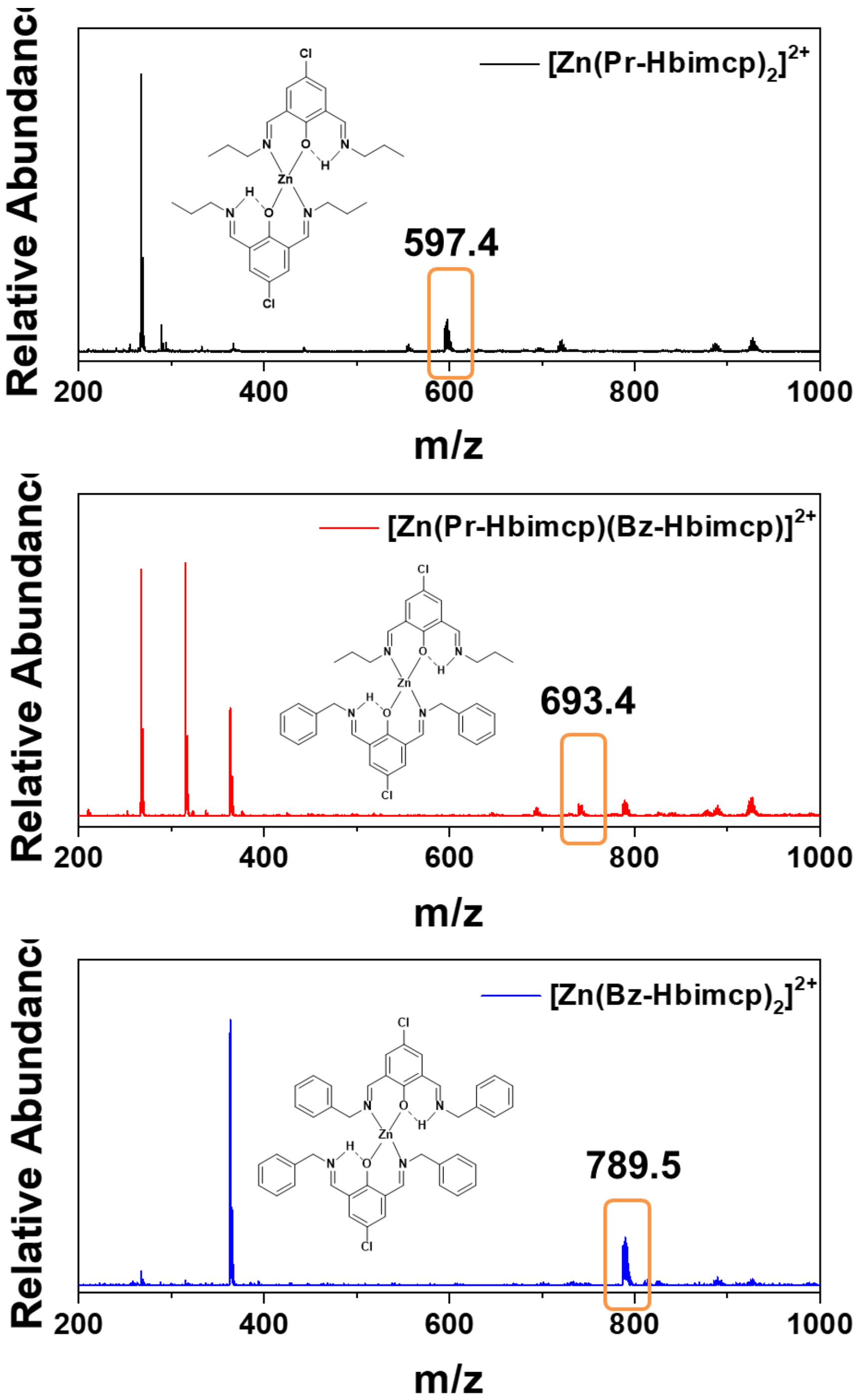

Supplementary Figure 1. ESI Mass spectrum for [Zn(Pr-Hbimcp)$_2$]$^{2+}$ (top), [Zn(Bz-Hbimcp)$_2$]$^{2+}$ (bottom) and mixture of [Zn(Pr-Hbimcp)$_2$]$^{2+}$ and [Zn(Bz-Hbimcp)$_2$]$^{2+}$ (middle).

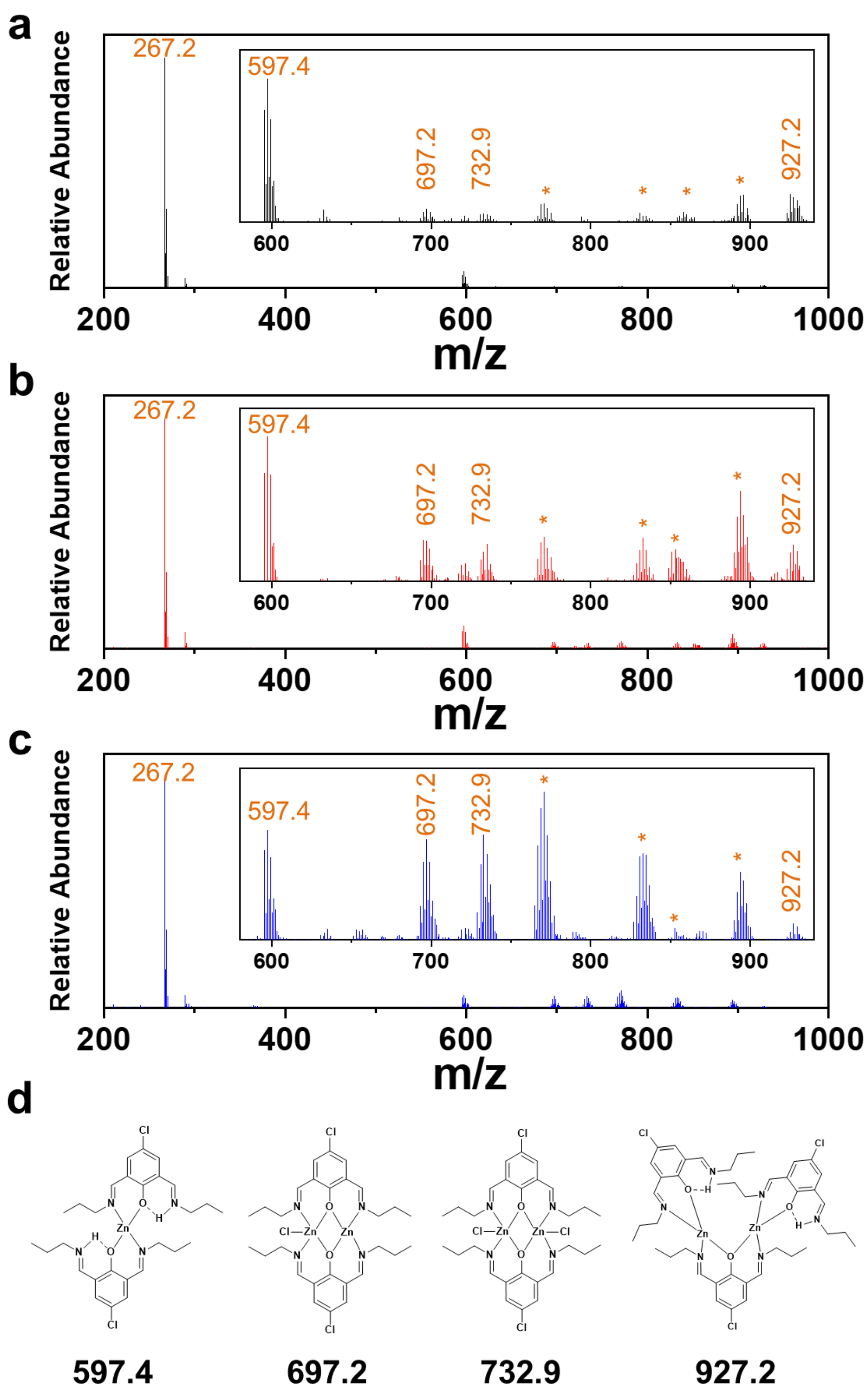

Supplementary Figure 2. ESI-MS spectra of the reaction product between Zn(II) complexes and Pr-Hbimcp ligand with different molar ratio. a, 2:1; b, 3:2; c, 1:1; d, the possible structure of the obtained peaks. The inserted figure is the partial enlargement of the main figure. The asterisks represent the peak that cannot be attributed.

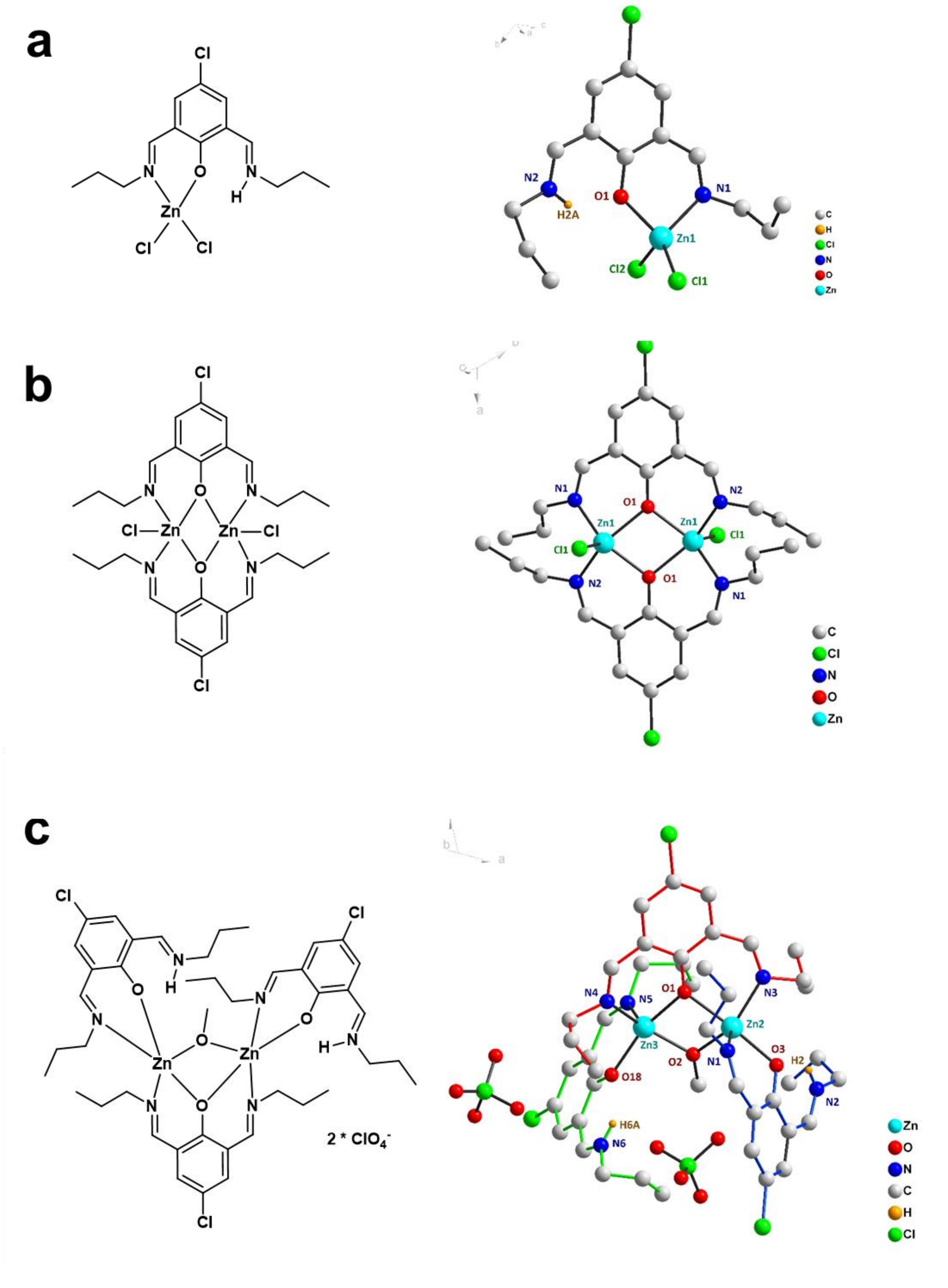

Supplementary Figure 3. Molecular and crystal structures of (a) Zn(Pr-Hbimcp)Cl_2, (b) Zn_2(Pr-bimcp)$_2Cl_2$ and (c) [Zn_2(Pr-bimcp)(Pr-Hbimcp)$_2$(CH_3O)](ClO_4)$_2$. Hydrogen atoms on carbon atoms are omitted for clarity.

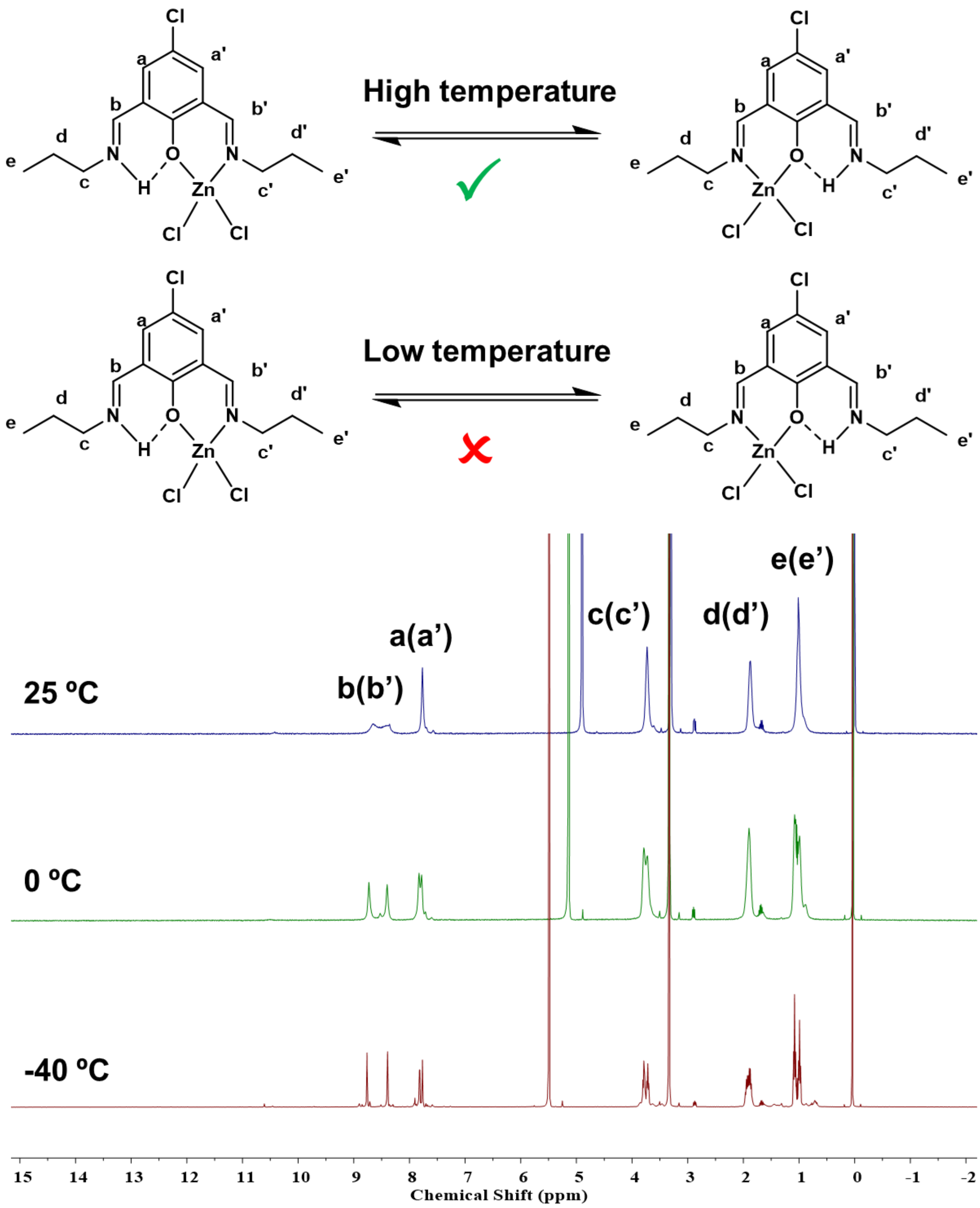

Supplementary Figure 4. Variable temperature ^{1}H NMR for Zn(Pr-Hbimcp)Cl_2 with the solvent of CD_3OD.

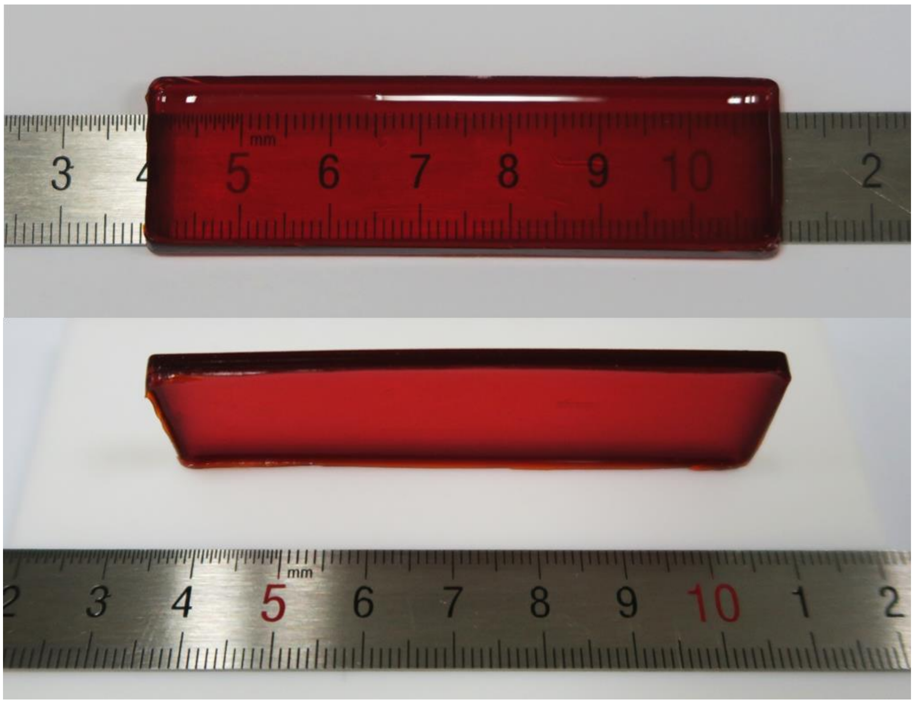

Supplementary Figure 5. The photo image of **Zn(Hbimcp)$_2$-PDMS** film (with a **Hbimcp-PDMS** ligand to Zn(II) metal molar ratio of 1:2).

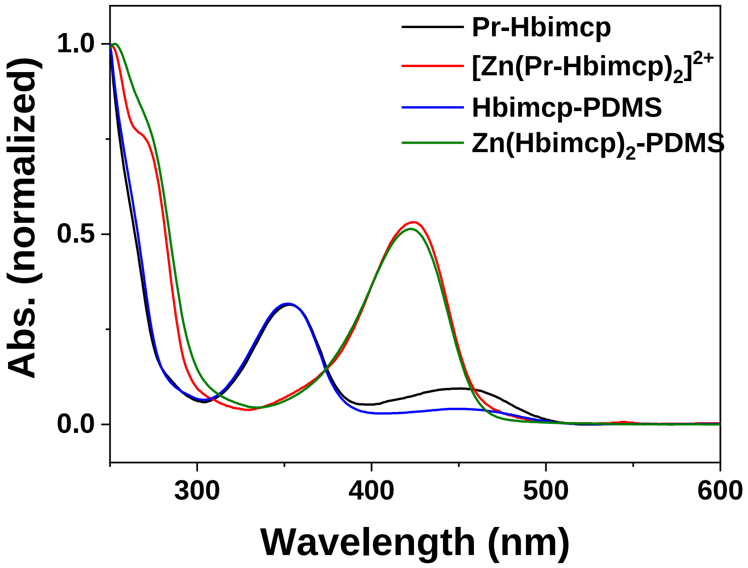

Supplementary Figure 6. UV-Vis spectra for **Pr-Hbimcp, Zn(Pr-Hbimcp)$_2$$^{2+}$**, **Hbimcp-PDMS** and **Zn(Hbimcp)$_2$-PDMS** polymers.

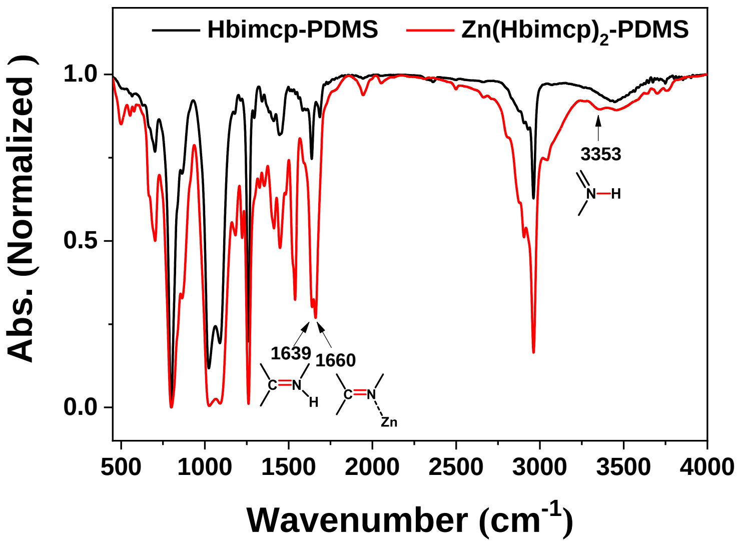

Supplementary Figure 7. IR spectra for **Hbimcp-PDMS** and **Zn(Hbimcp)$_2$-PDMS** polymers.

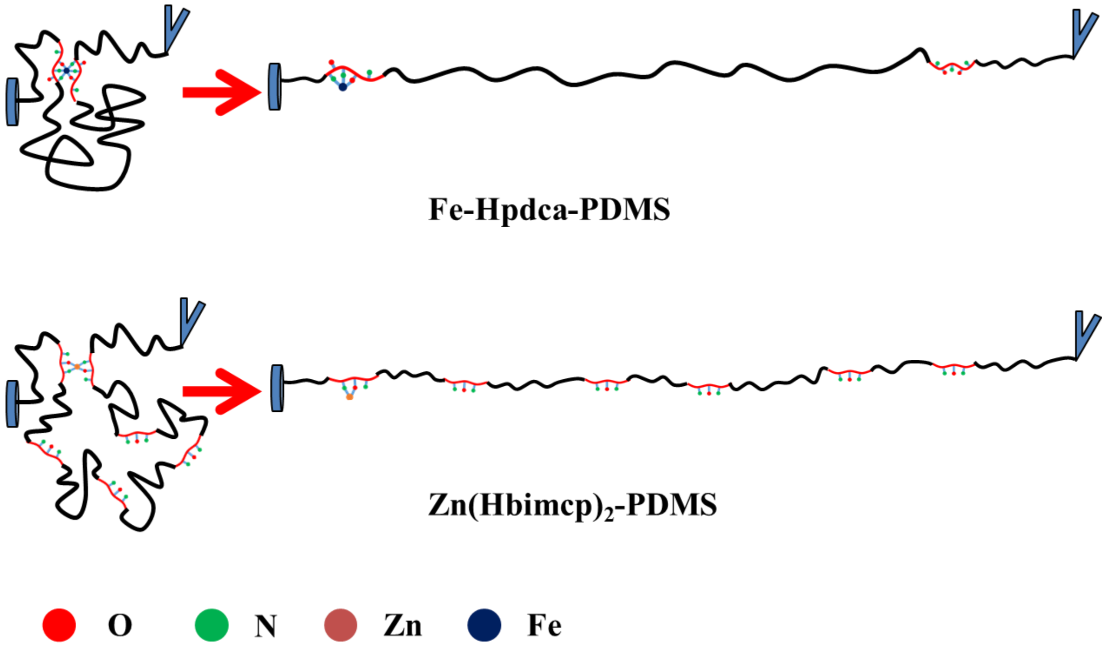

Supplementary Figure 8. The different folding modes for Fe-Hpdca-PDMS and **Zn(Hbimcp)$_2$-PDMS** polymers.

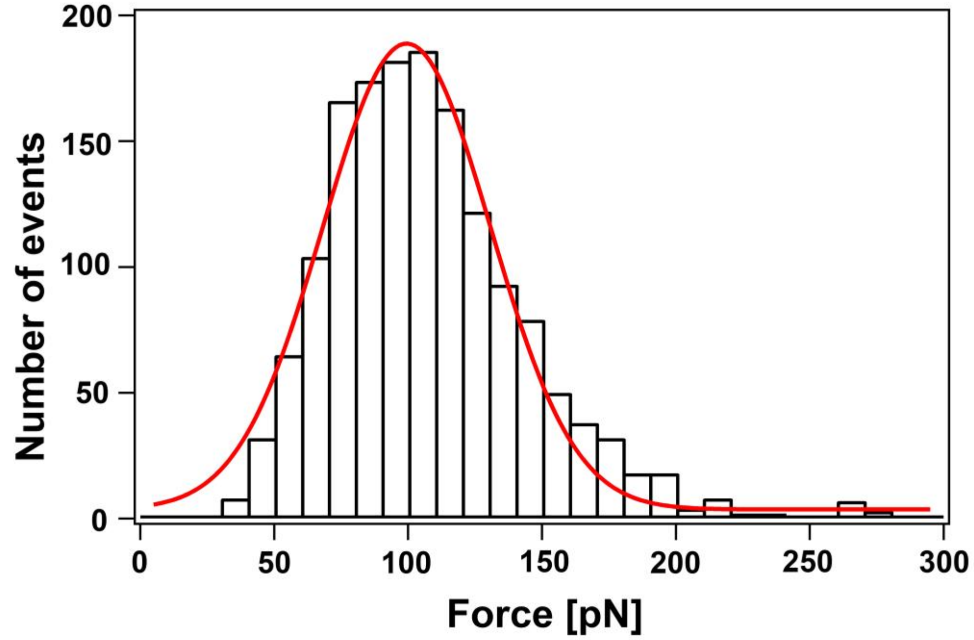

Supplementary Figure 9. Histogram (n = 1566) of the rupture forces of the $[Zn(Hbimcp)_2]^{2+}$ coordination complexes.

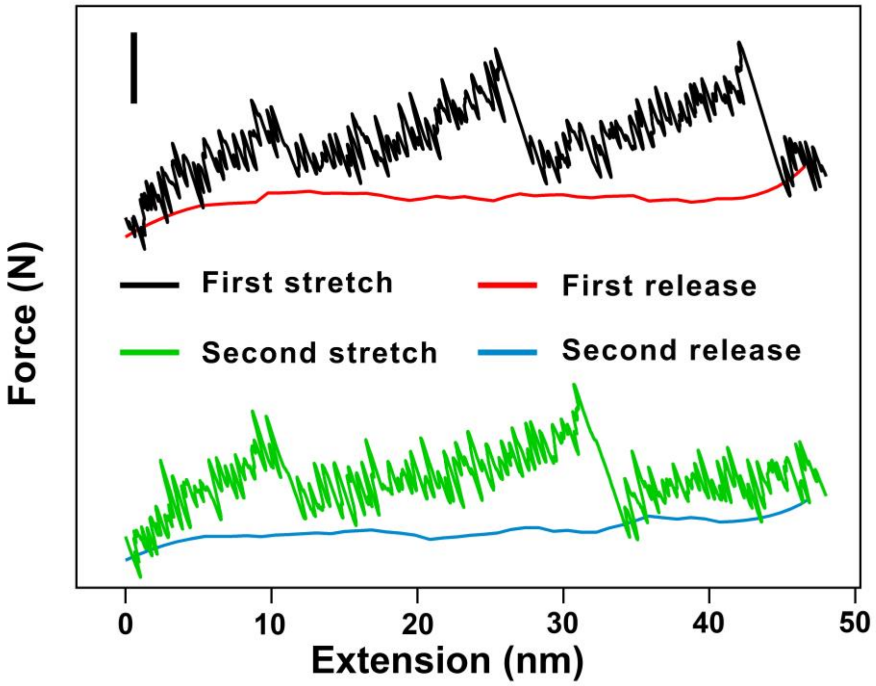

Supplementary Figure 10. Typical force–extension curves of **$Zn(Hbimcp)_2$-PDMS** from stretching–releasing cycles that show reversible unfolding and refolding. Scale bar for the vertical axis, 50 pN.

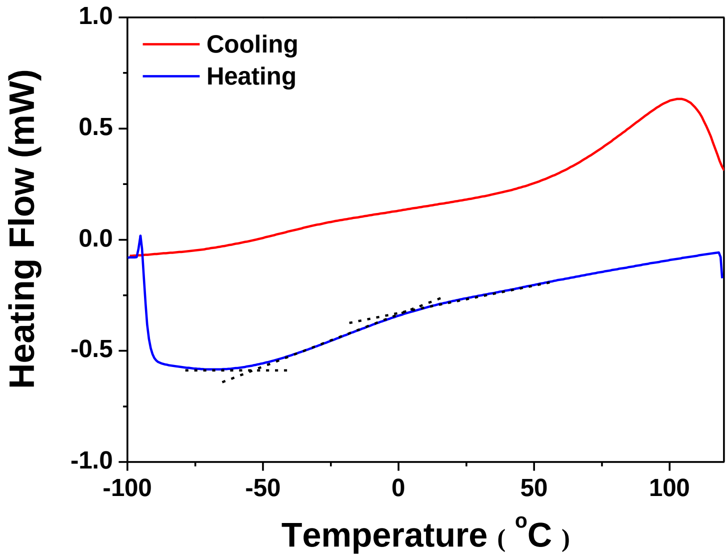

Supplementary Figure 11. The DSC curves of **Zn(Hbimcp)$_2$-PDMS** polymer **(**with the Zn(II) metal to **Hbimcp-PDMS** ligand molar ratio of 1:2**).**

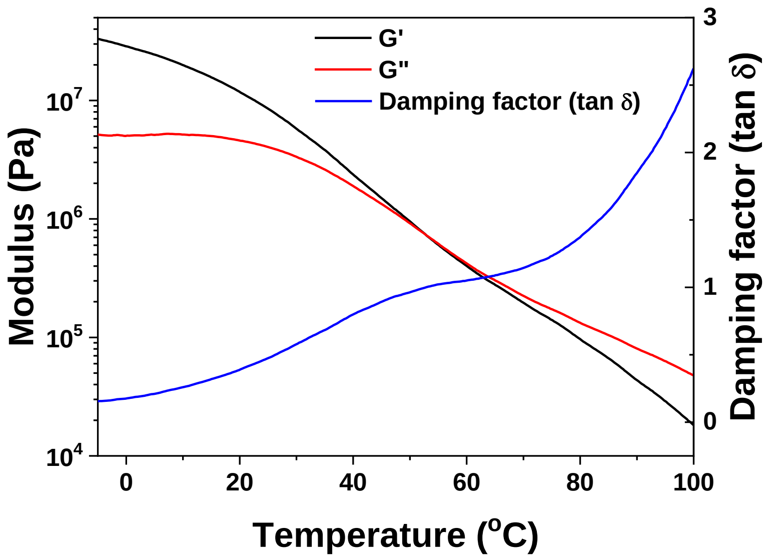

Supplementary Figure 12. Temperature sweeping rheological test of **Zn(Hbimcp)$_2$-PDMS** polymer (with the Zn(II) metal to **Hbimcp-PDMS** ligand molar ratio of 1:2).

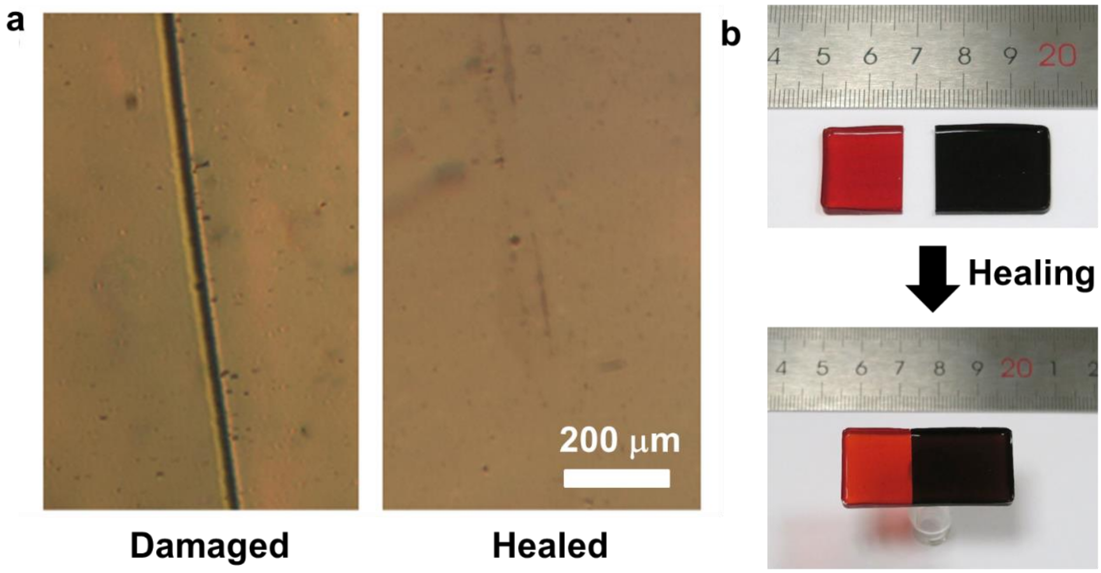

Supplementary Figure 13. Microscopic images (a) and optical images (b) of two pieces of **Zn(Hbimcp)$_2$-PDMS** film when damaged and healed at room temperature.

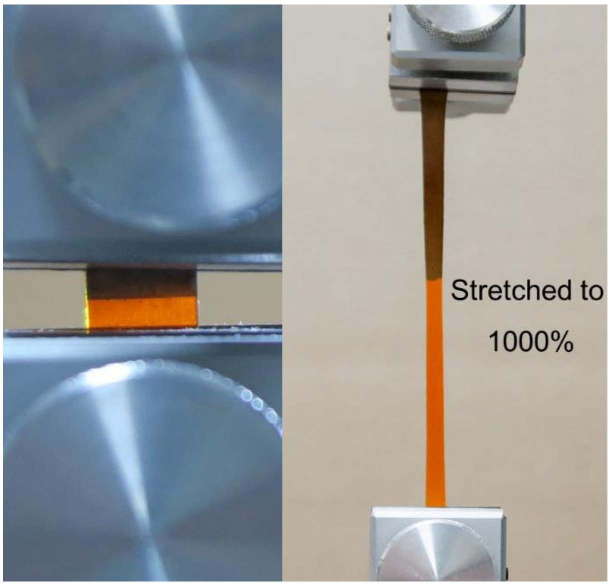

Supplementary Figure 14. Optical images of the healed film under a large strain.

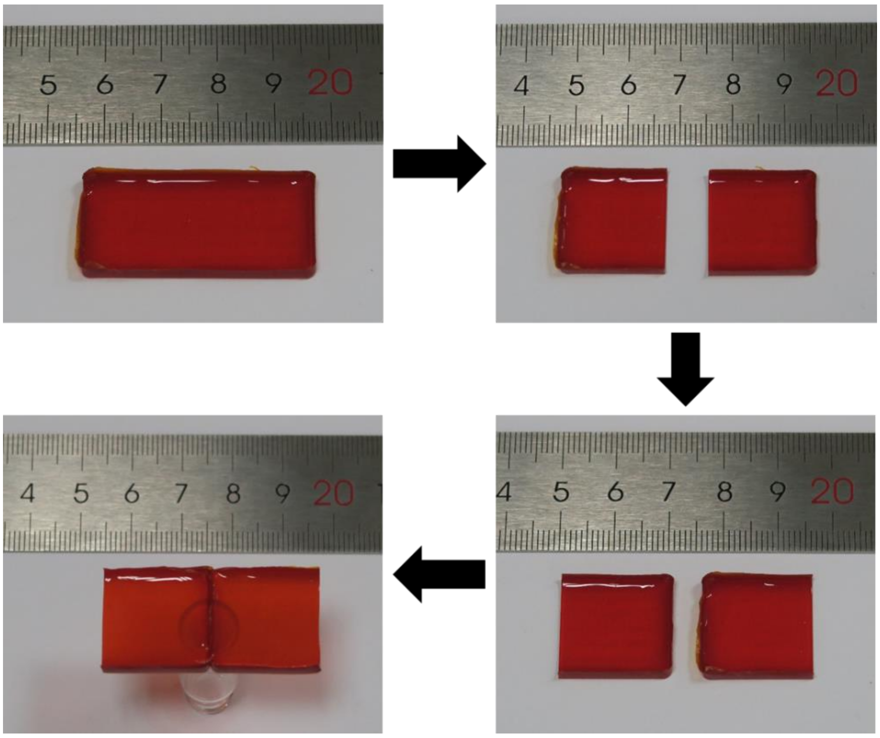

Supplementary Figure 15. Optical images of the Self-healing of **Zn(Hbimcp)$_2$-PDMS** film (with a Zn(II) metal to **Hbimcp-PDMS** ligand molar ratio of 1:2) at undamaged surface.

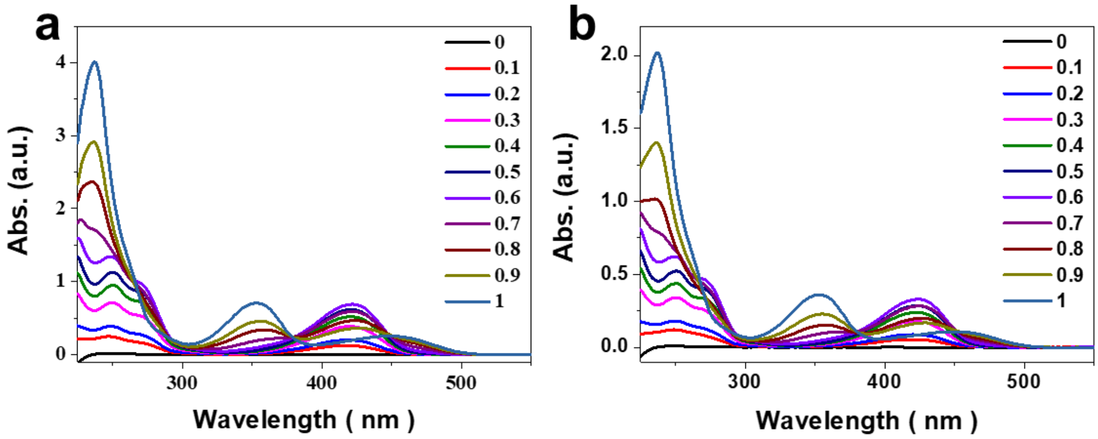

Supplementary Figure 16. UV-Vis spectra of **Pr-Hbimcp** and Zn(II) complex solutions with the different original solution concentration(a. 10^{-3} M; b, 5×10^{-4} M) and ligand and metal ion ratio (from 0.0 to 1.0).

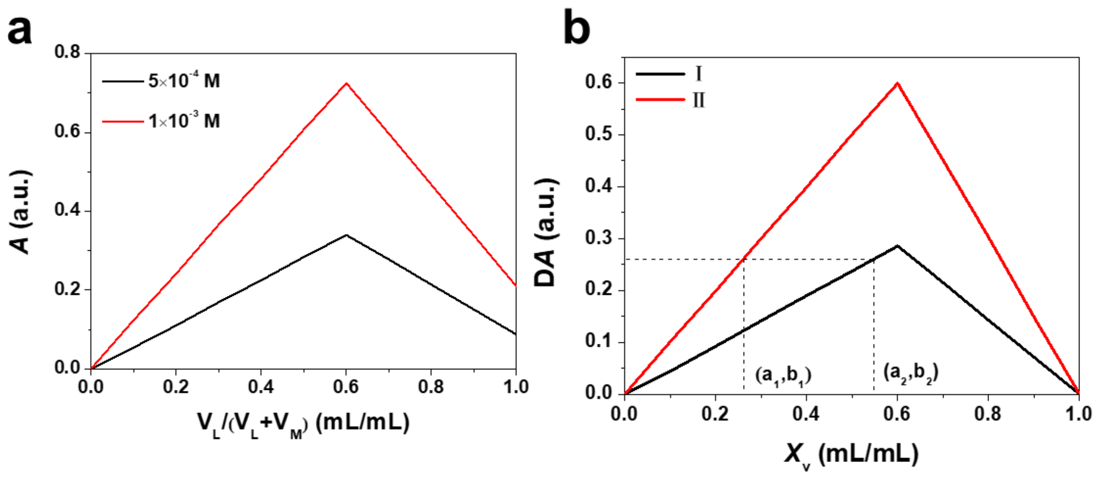

Supplementary Figure 17. UV-Vis absorption values (λ_{max}=422 nm) of [Zn(Pr-Hbimcp)$_2$]$^{2+}$complex solution at different $V_L/(V_L+V_M)$ ratios. **a**, Original absorption values; **b**, calibrate absorption values.

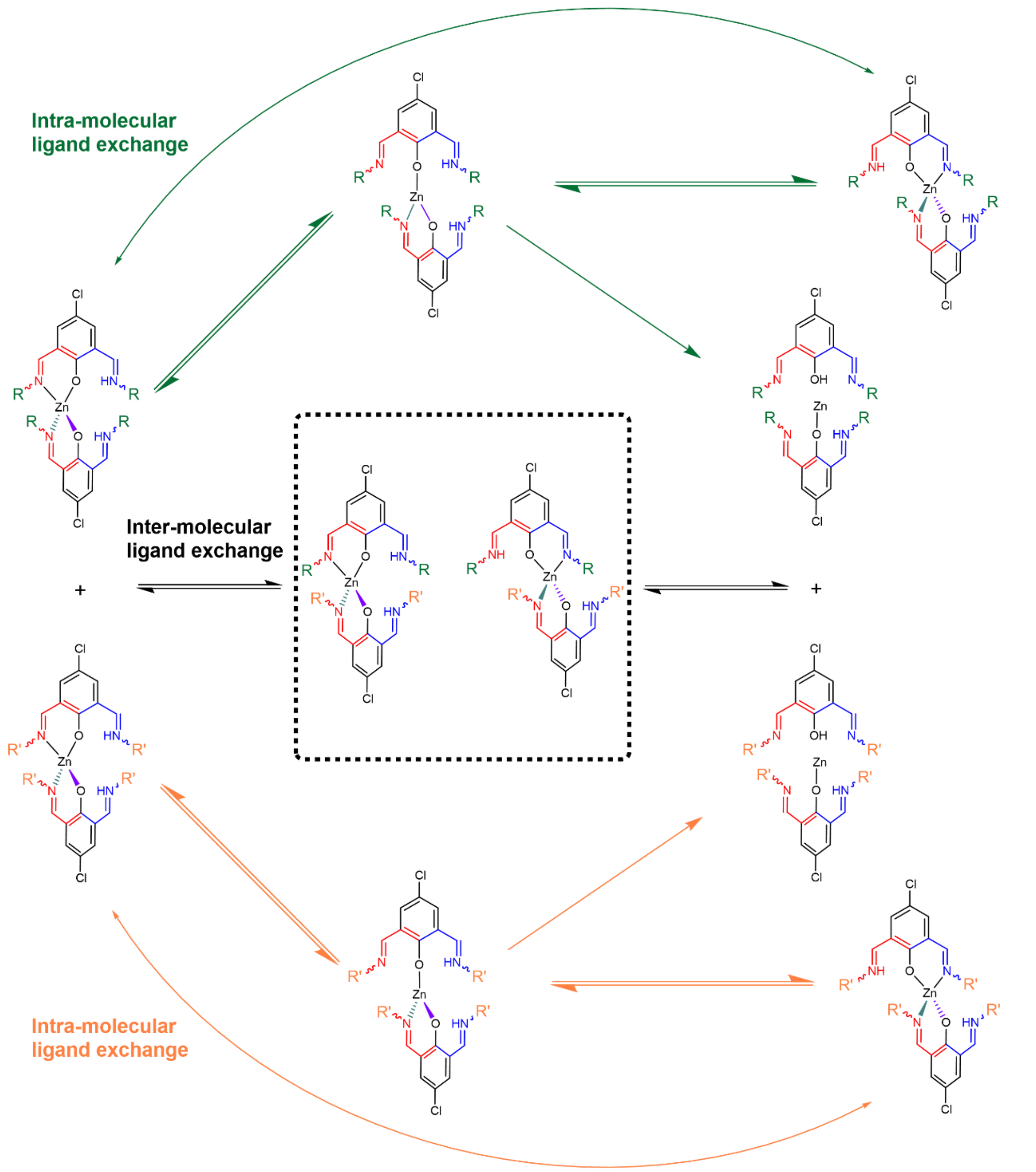

Supplementary Figure 18. The intra-molecular and inter-molecular ligand exchange process in Zn-Hbimcp complexes.

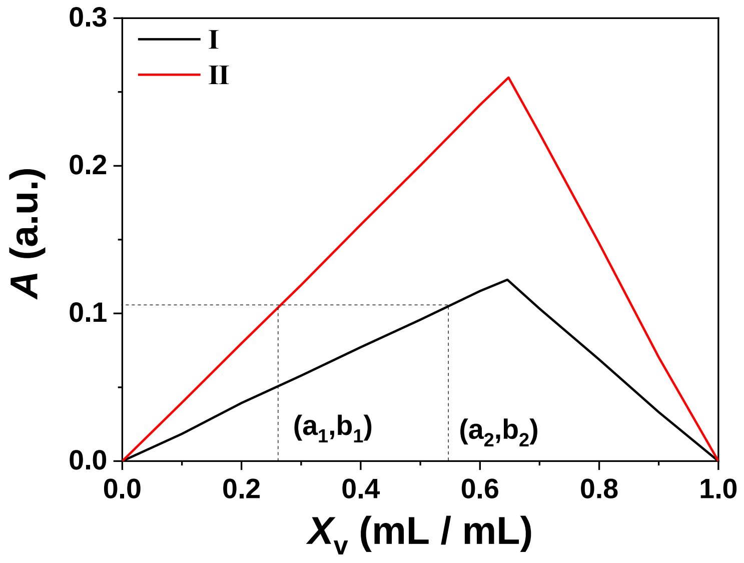

Supplementary Figure 19. Calibrate UV-Vis absorption values (λ_{max} = 399 nm) of $[Ni(Pr\text{-}Hbimcp)_2]^{2+}$complex solution at different $V_L/(V_L+V_M)$ ratios.

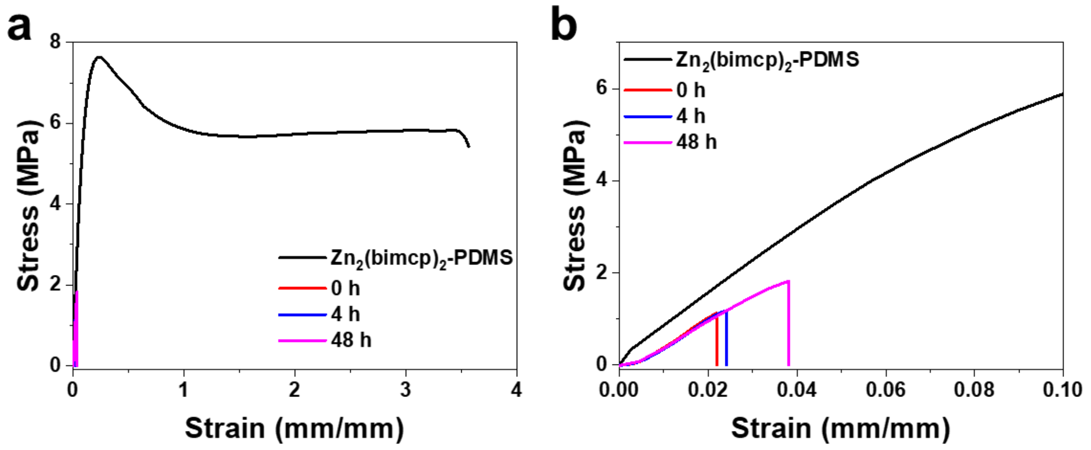

Supplementary Figure 20. The self-healing properties of **$Zn_2(bimcp)_2$-PDMS** at 25 °C. **b** is the partial enlargement of **a**. The **$Zn_2(bimcp)_2$-PDMS** polymer was partially damaged (remained 10% uncut) to keep the incision better contact.

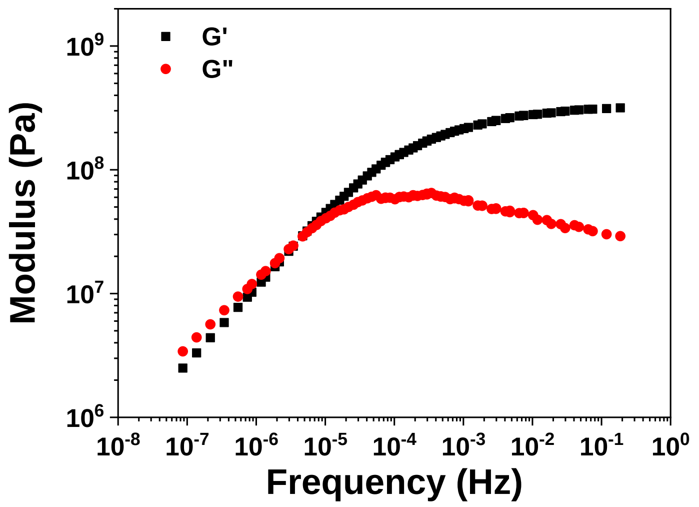

Supplementary Figure 21. The time–temperature superposition curve of **$Zn_2(bimcp)_2$-PDMS** polymer at 25 °C.

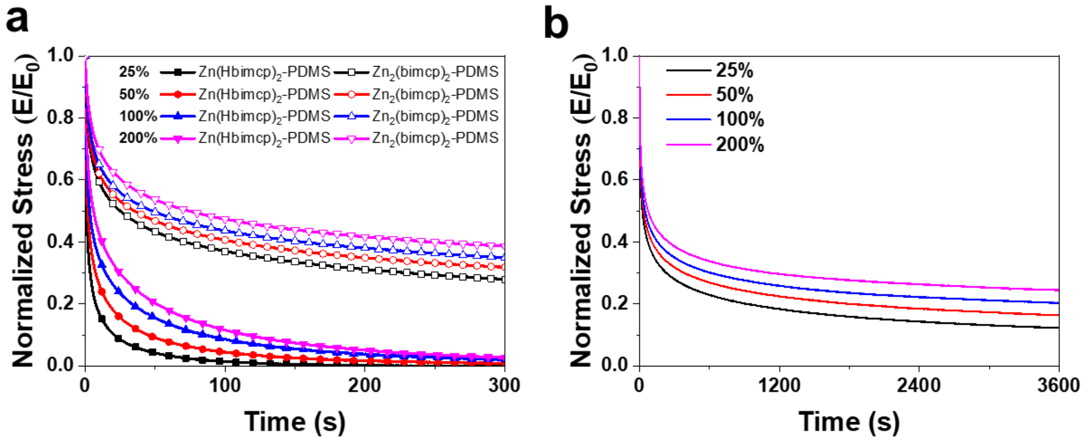

Supplementary Figure 22. The stress-relaxation curves of **$Zn_2(bimcp)_2$-PDMS** at different initial strain, which indicated there was no obvious ligand exchange phenomenon in the polymer of $Zn_2(bimcp)_2$-PDMS within 1 h. a, compare with the curves of **$Zn(Hbimcp)_2$-PDMS** with the duration time of 300 s. b, with the duration time of 3600s.

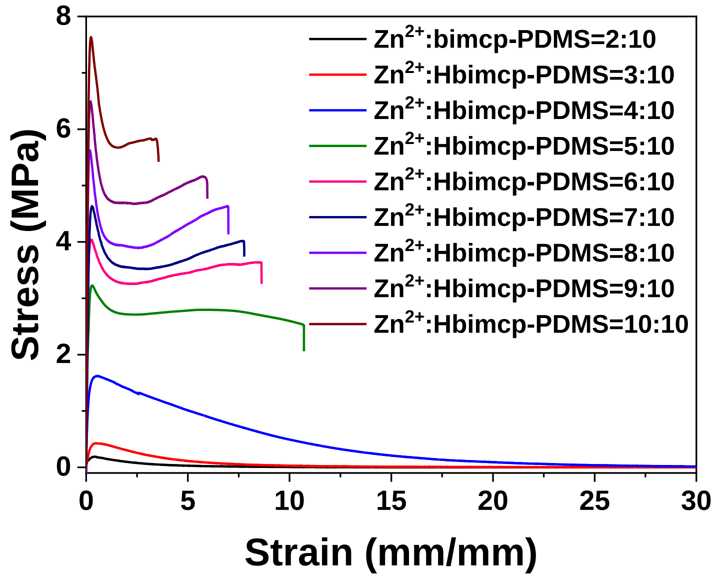

Supplementary Figure 23. Strain-stress curves of films prepared with different molar ratios of Zn(II) metal to **Hbimcp-PDMS** ligand.

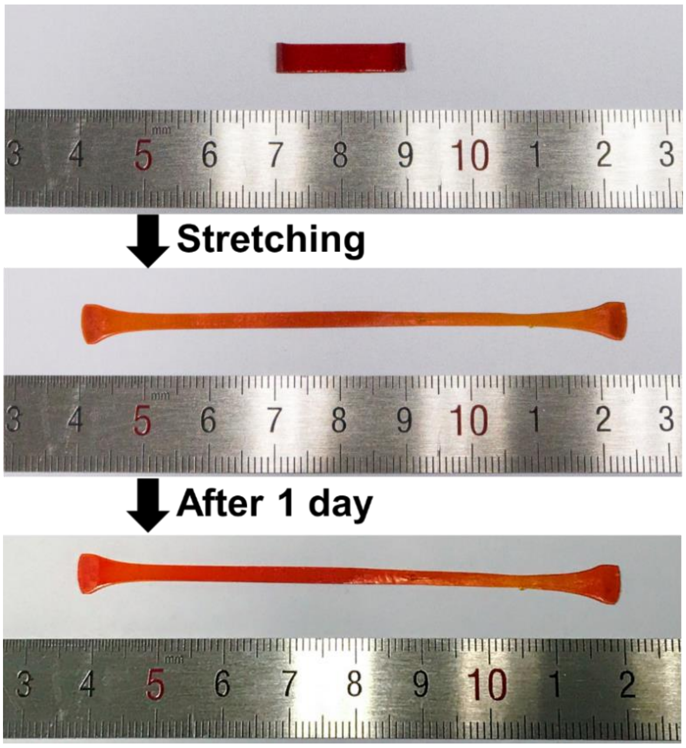

Supplementary Figure 24. The configuration keeping property of the stretched **Zn(Hbimcp)$_2$-PDMS** polymer.

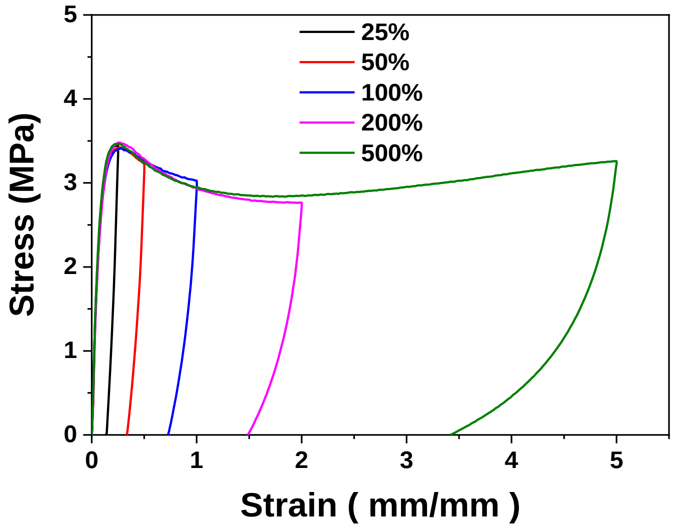

Supplementary Figure 25. Cyclic strain-stress curves of the **Zn(Hbimcp)$_2$-PDMS** film with different tensile strain (from 25 to 500%).

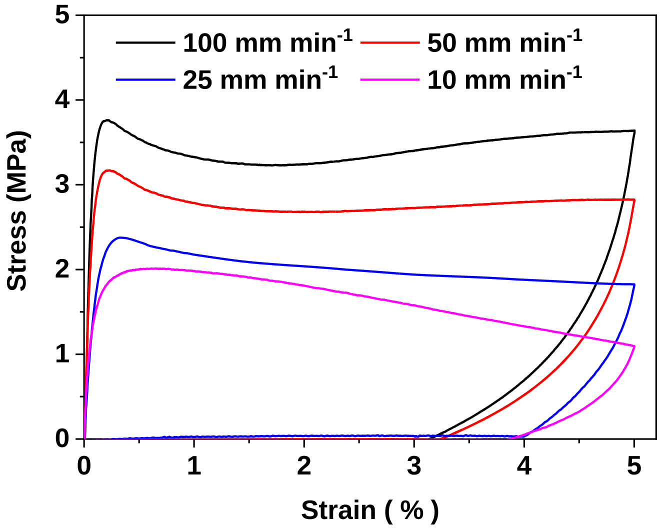

Supplementary Figure 26. Cyclic strain-stress curves of the **Zn(Hbimcp)$_2$-PDMS** film with different strain rate.

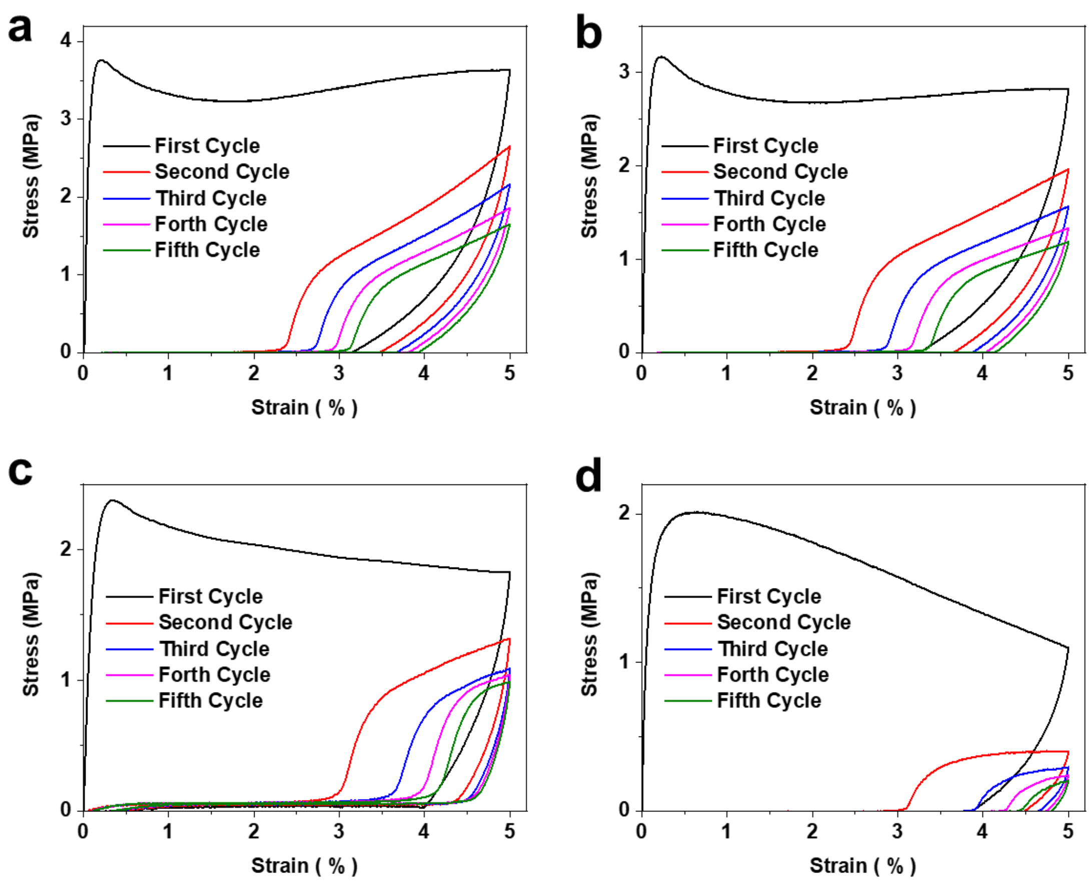

Supplementary Figure 27. Cyclic strain-stress curves of the **Zn(Hbimcp)$_2$-PDMS** film with multiple cycles and different strain rate. a, 100 mm min^{-1}. b, 50 mm min^{-1}. c, 25 mm min^{-1}. d, 10 mm min^{-1}.

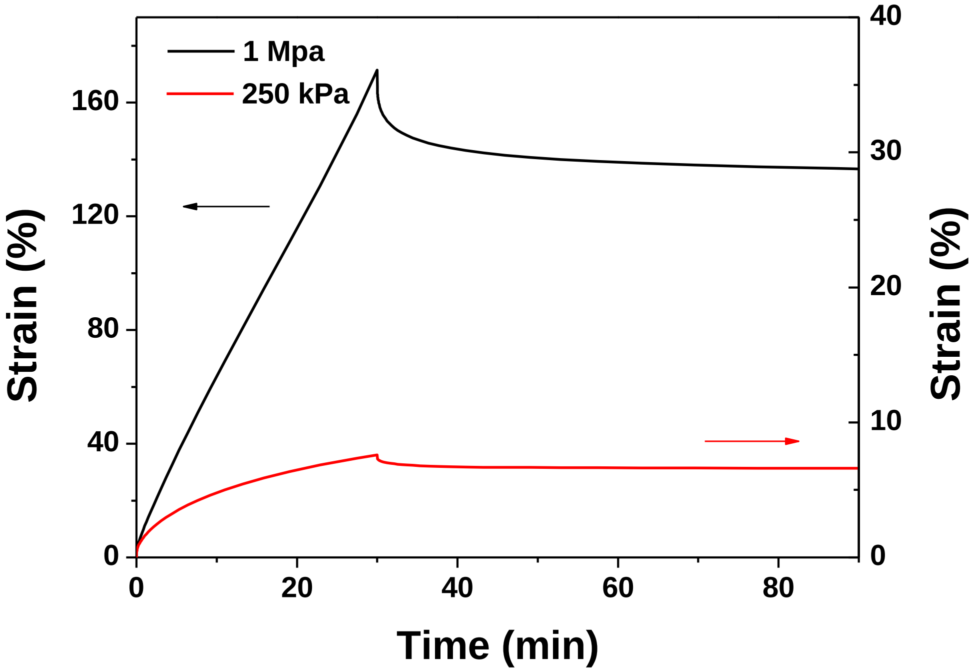

Supplementary Figure 28. Creep-recovery curves of **Zn(Hbimcp)$_2$-PDMS** polymer under constant strain stress (1 MPa and 250 kPa, respectively).

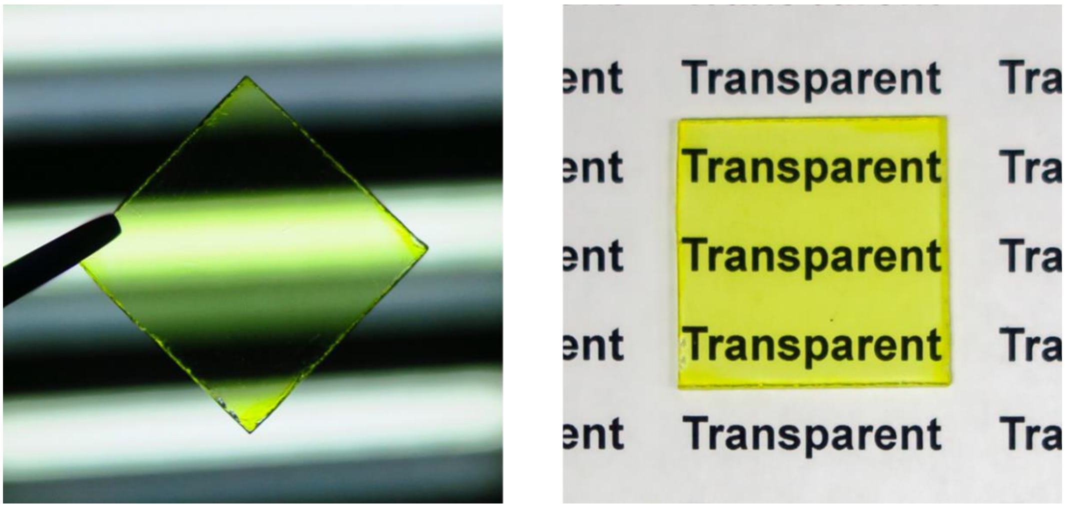

Supplementary Figure 29. The film-forming property of **Zn(Hbimcp)$_2$-PDMS.**

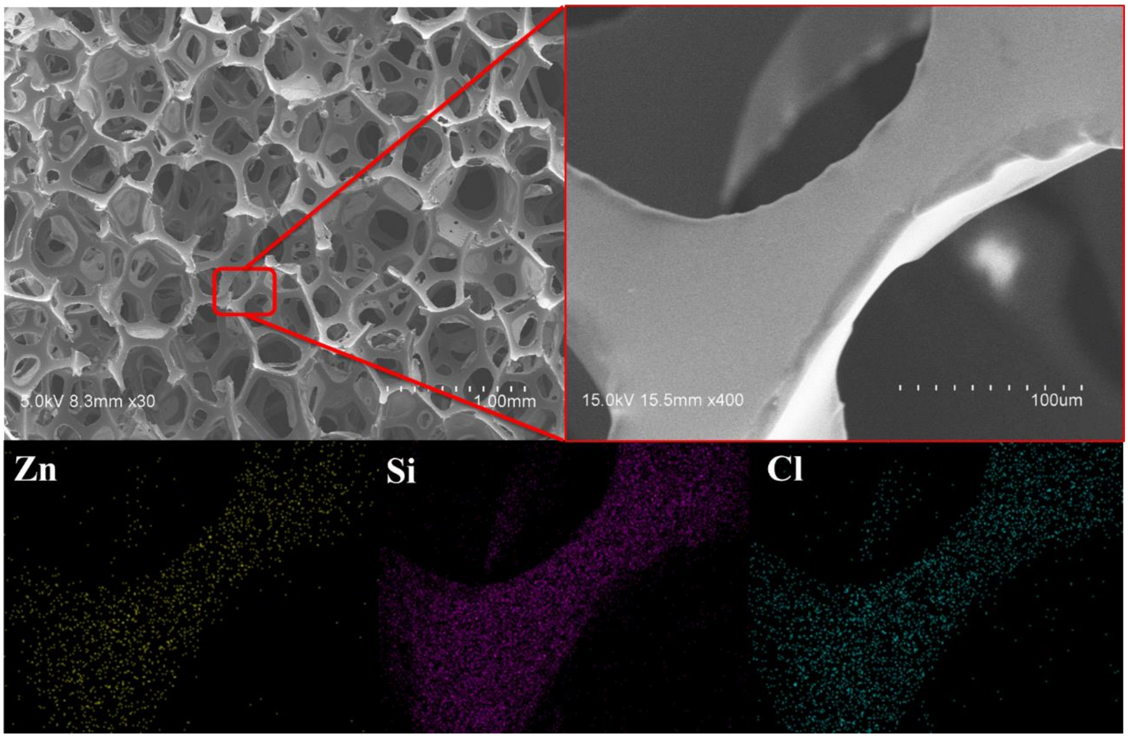

Supplementary Figure 30. SEM and EDX spectra of **Zn(Hbimcp)$_2$-PDMS** coated sponge.

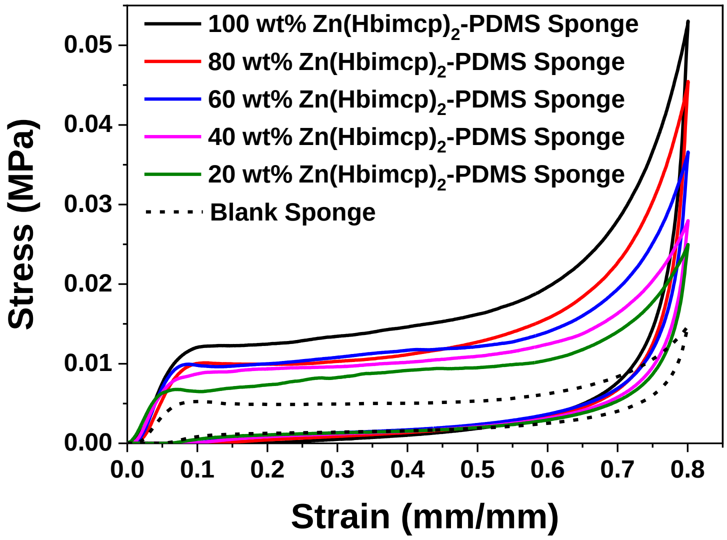

Supplementary Figure 31. Cyclic strain-stress curves of composite sponge with different quality fraction of coated **Zn(Hbimcp)$_2$-PDMS** at strain rate of 50 mm min^{-1}.

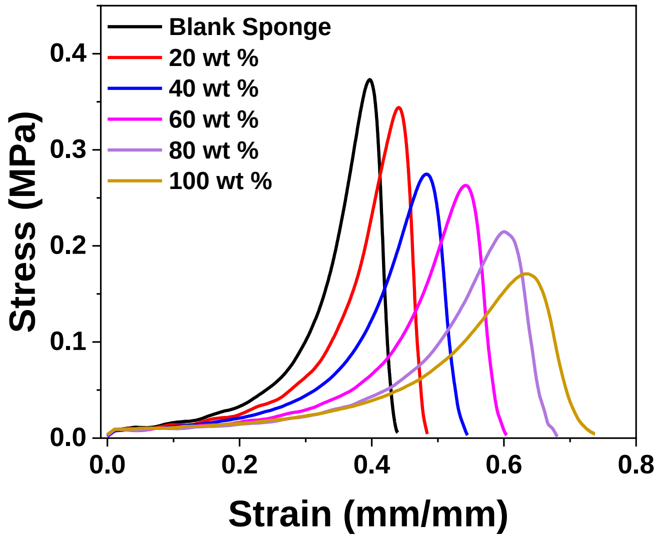

Supplementary Figure 32. Strain-stress curves of composite sponge with different weight ratio of coated **Zn(Hbimcp)$_2$-PDMS** under the dynamic compressing tests (drop impact tests). It shows that with increasing the weight ratio of **Zn(Hbimcp)$_2$-PDMS**, the composite sponge shows higher strain of impact and lower stress of impact, which indicates that coating **Zn(Hbimcp)$_2$-PDMS** onto sponges decreased the damage resistance but increased the damage tolerance.

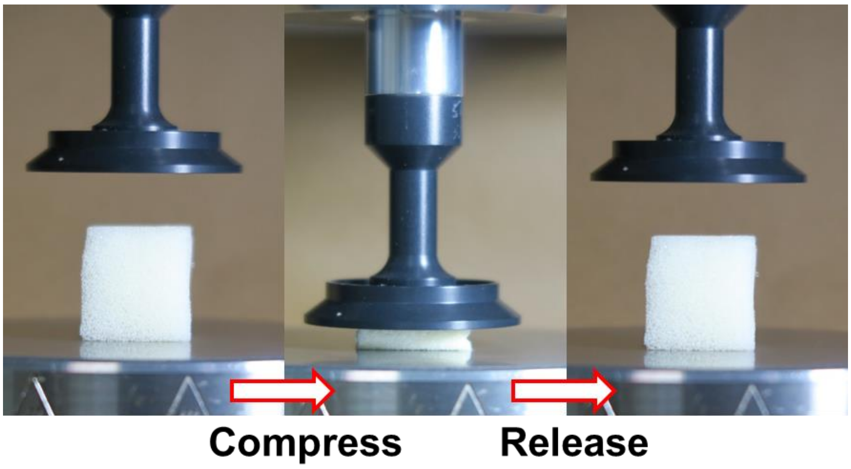

Supplementary Figure 33. Compression and recovery processes of blank sponge.

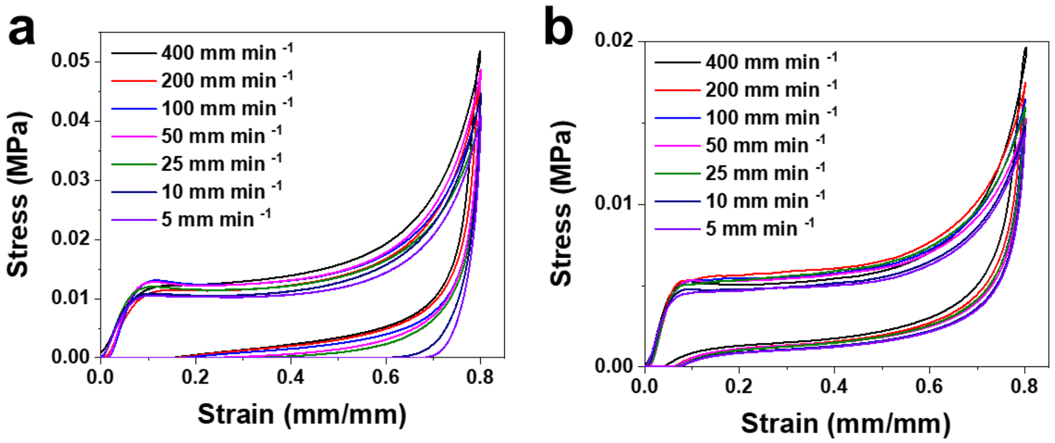

Supplementary Figure 34. Cyclic strain-stress curves of the **Zn(Hbimcp)$_2$-PDMS** coated sponge (a) and blank sponge (b) with different strain rate. It shows that the residual strain and the configuration recovery time of **Zn(Hbimcp)$_2$-PDMS** coated sponge will decrease when the compression speed is higher.

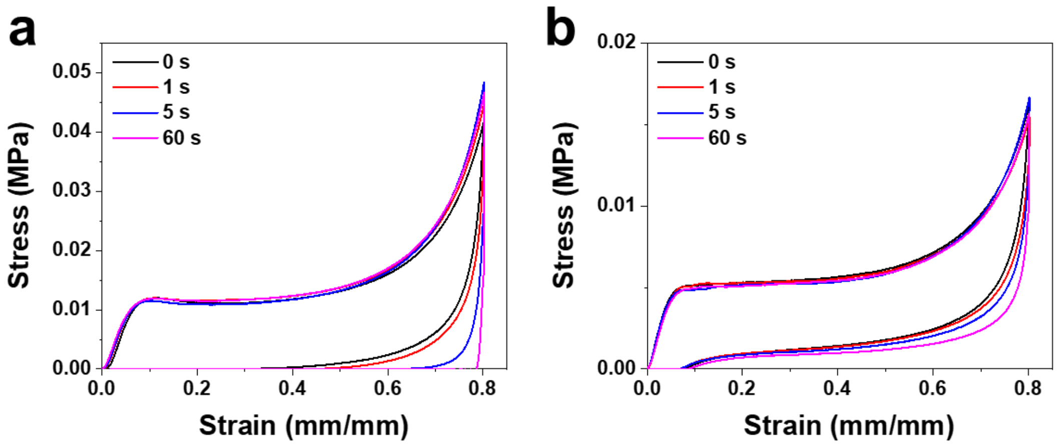

Supplementary Figure 35. Cyclic strain-stress curves of the **Zn(Hbimcp)$_2$-PDMS** coated sponge (a) and blank sponge (b) with different duration time at full compression. It shows that the residual strain and the configuration recovery time of **Zn(Hbimcp)$_2$-PDMS** coated sponge will decrease when the duration time at full compression is shorter.

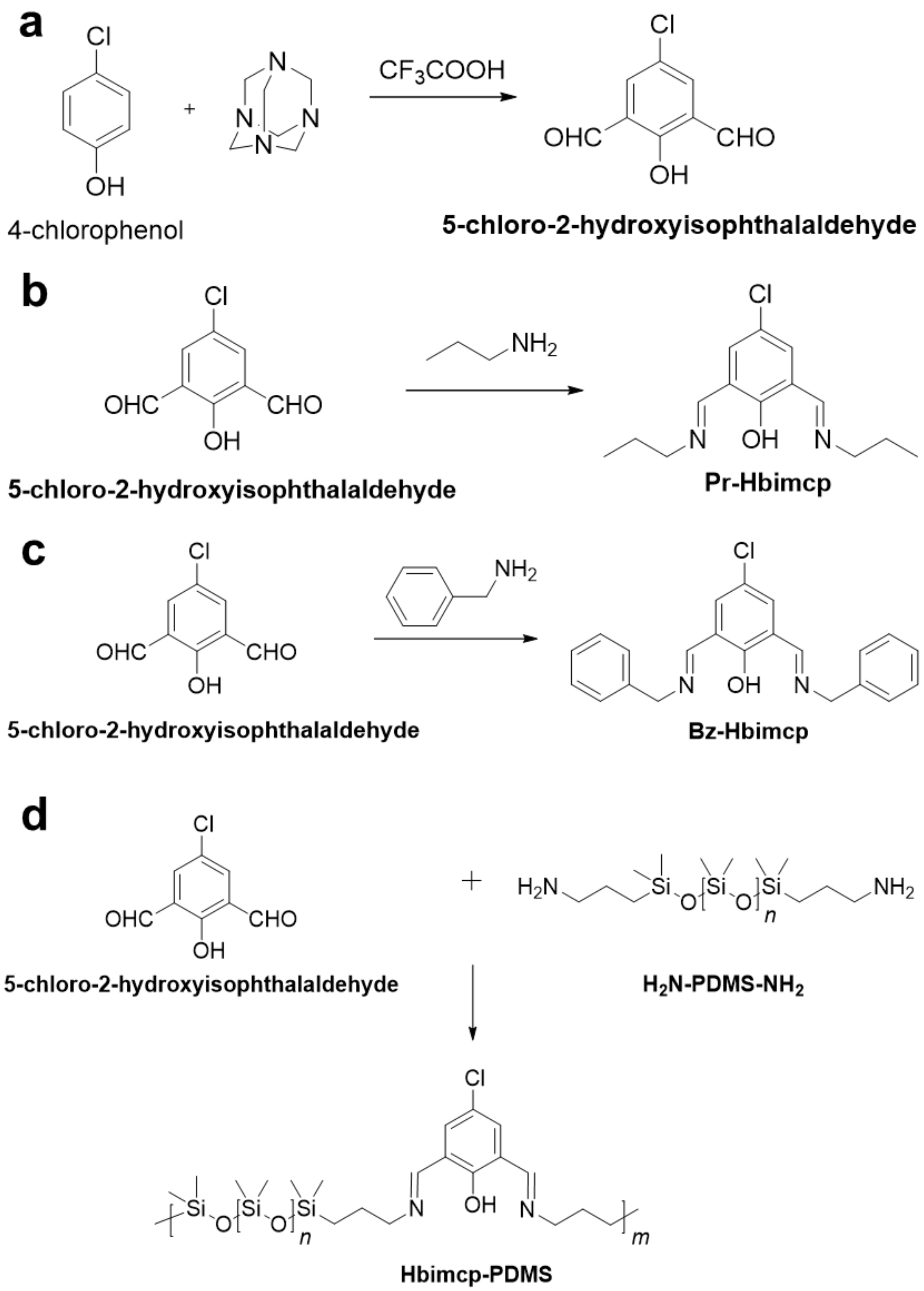

Supplementary Figure 36. Synthesis of small molecule ligands and polymer ligands. a, Synthesis of 5-chloro-2-hydroxyisophthalaldehyde. b, Synthesis of 2,6-bis((propylimino)methyl)-4-chlorophenol. c, Synthesis of 2,6-bis((benzylimino)methyl) -4-chlorophenol. d, Synthesis of Hbimcp-PDMS ligand.

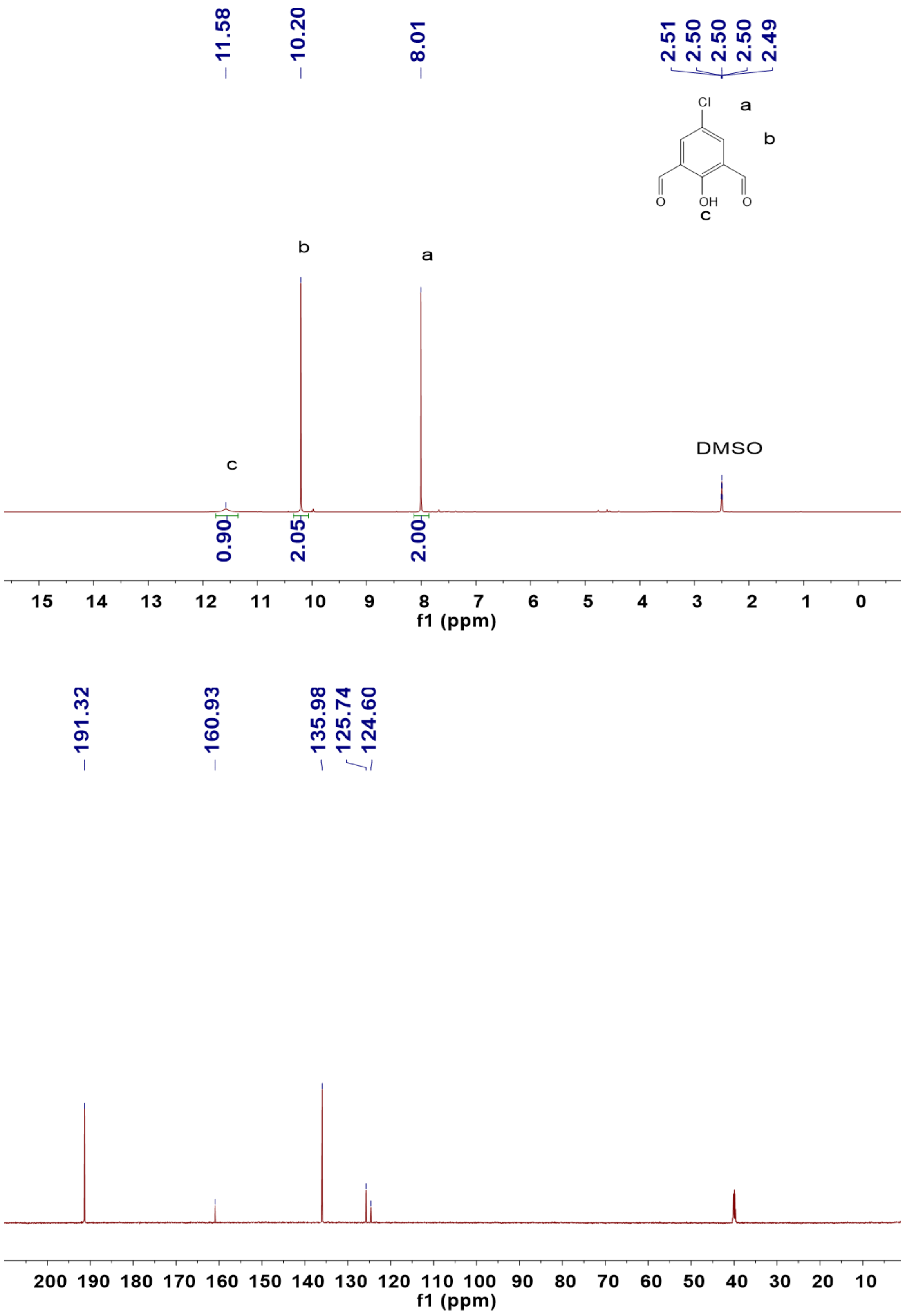

Supplementary Figure 37. ^{1}H NMR (top) and ^{13}C NMR (bottom) spectra of 5-chloro-2-hydroxyisophthalaldehyde.

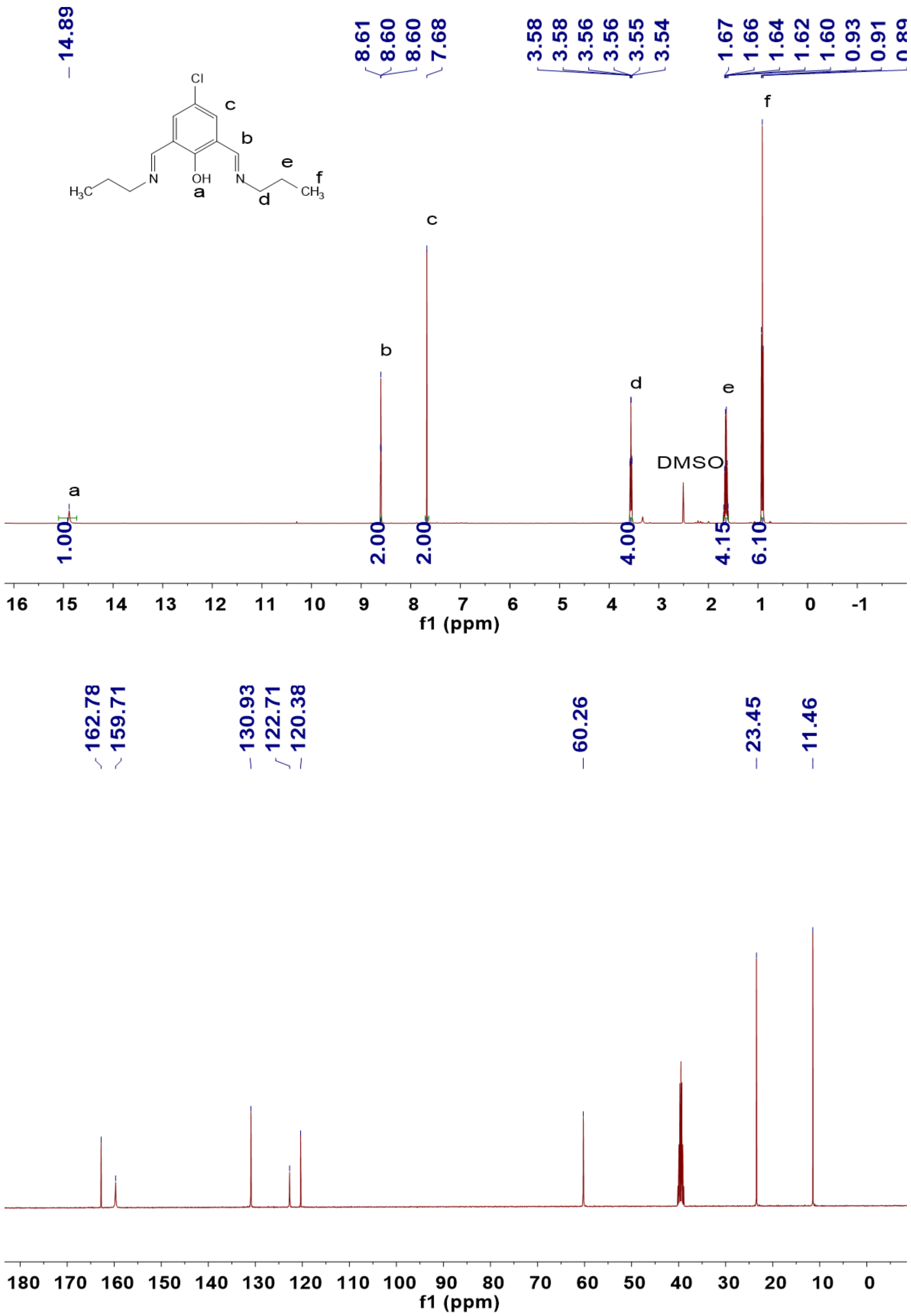

Supplementary Figure 38. ^{1}H NMR (top) and ^{13}C NMR (bottom) spectra of **Pr-Hbimcp.**

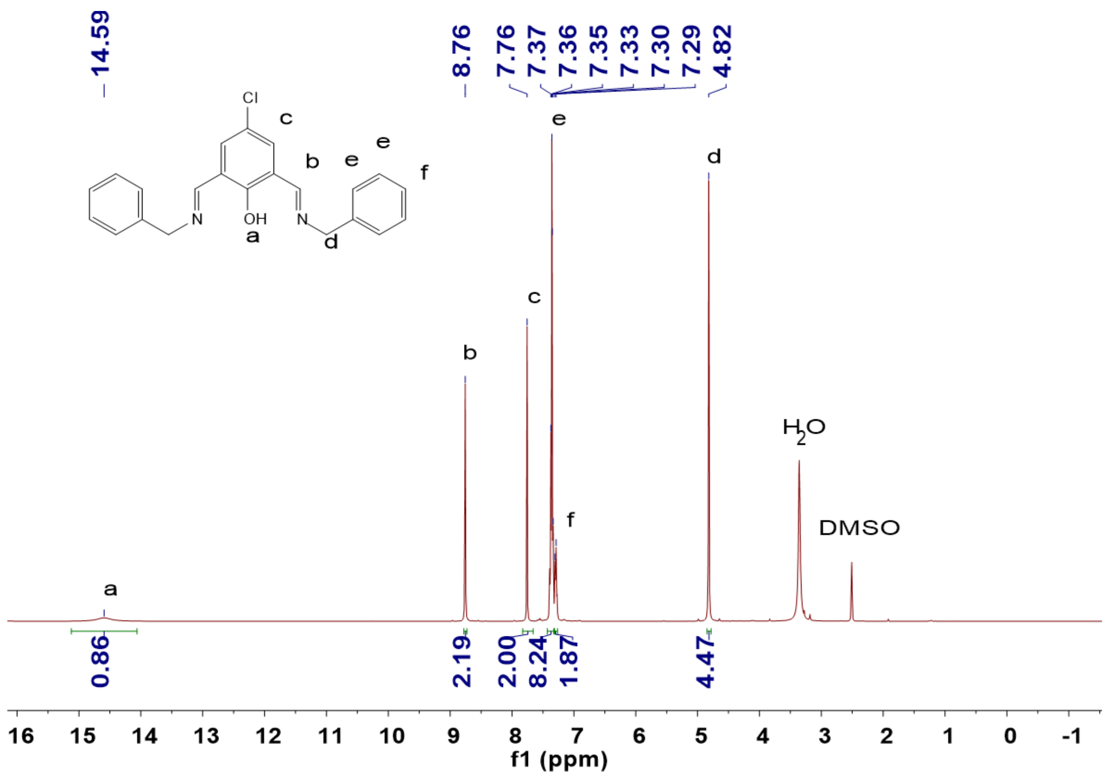

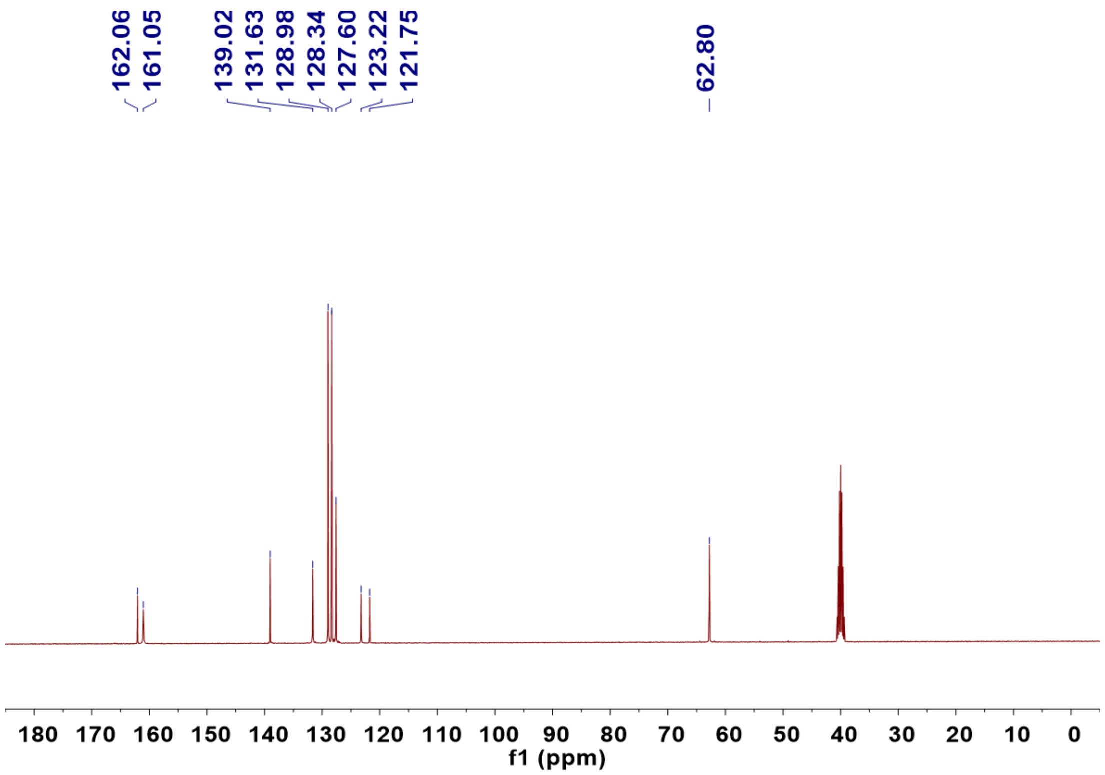

Supplementary Figure 39. ^{1}H NMR (top) and ^{13}C NMR (bottom) spectra of **Bz-Hbimcp.**

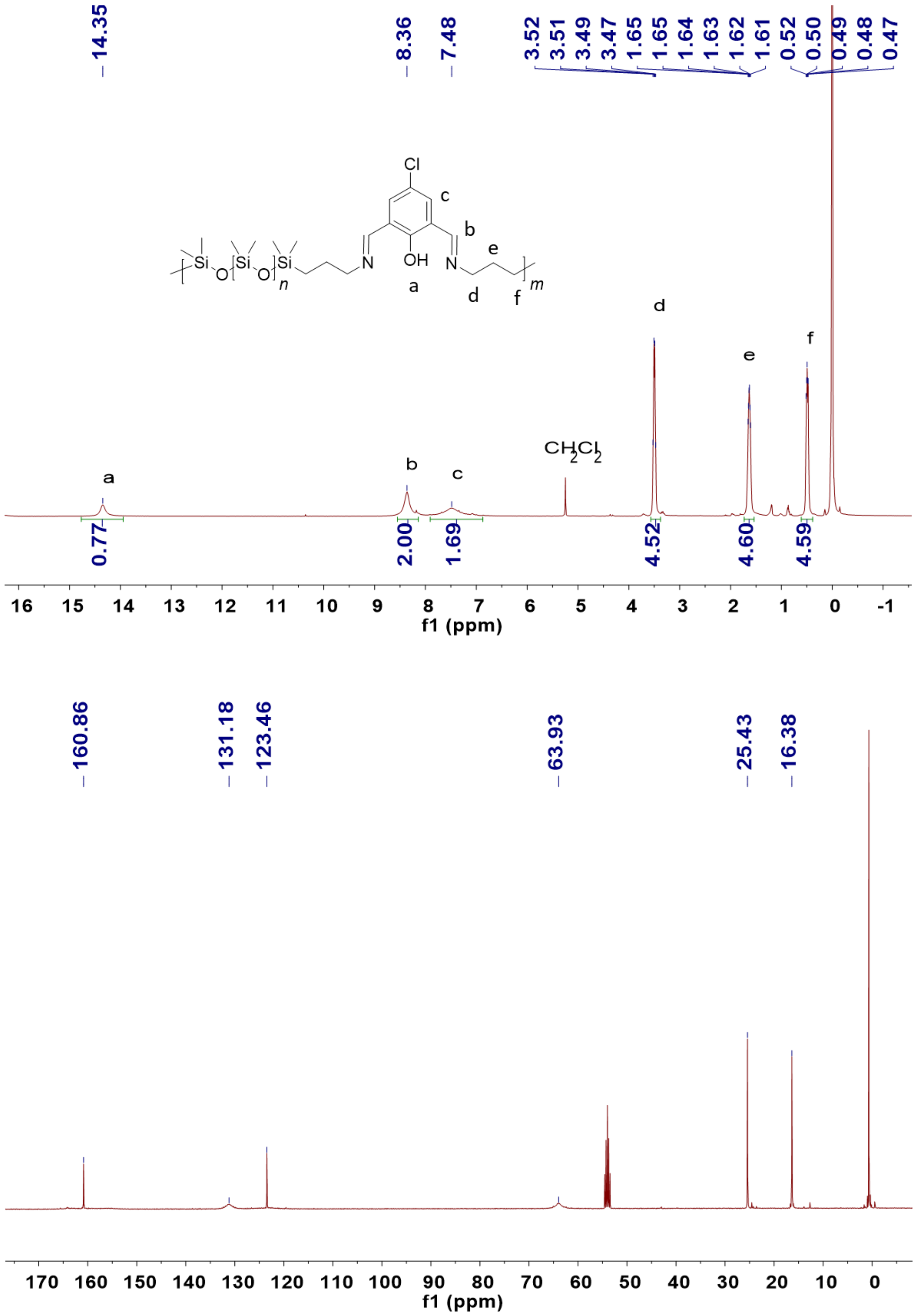

Supplementary Figure 40. ^{1}H NMR (top) and ^{13}C NMR (bottom) spectra of **Hbimcp-PDMS** ligand.

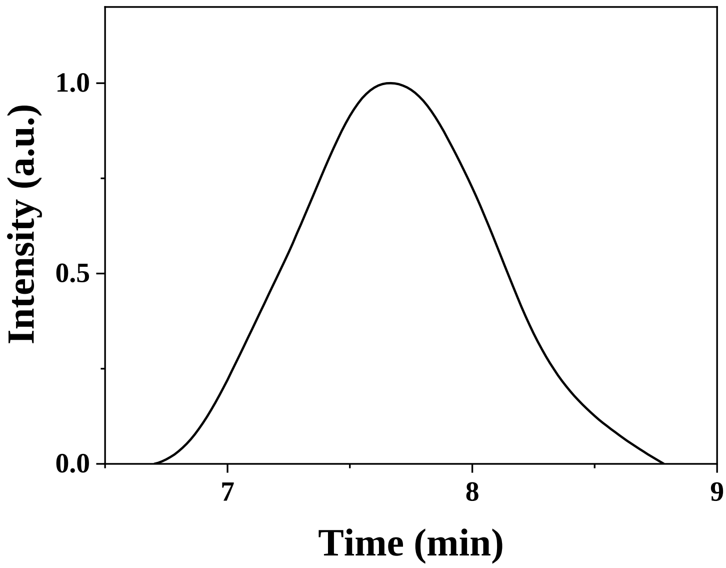

Supplementary Figure 41. GPC elution curves of Hbimcp-PDMS.

Supplementary Table 1. Healing efficiencies of **Zn(Hbimcp)$_2$-PDMS** with various healing time.

Healing time	Maximal strength (MPa)	Breaking strength (MPa)	Breaking strain (mm mm^{-1})	Healing efficiency (%)
Original	3.22	2.52	10.71	-
24h	3.15 ± 0.34	2.42 ± 0.27	10.60 ± 0.80	98.9 ± 1.9
12h	2.88 ± 0.26	2.44 ± 0.21	9.17 ± 0.67	85.6 ± 2.4
6h	2.82 ± 0.29	2.40 ± 0.35	8.24 ± 0.73	76.9 ± 3.7
3h	2.72 ± 0.32	2.26 ± 0.31	6.17 ± 0.58	57.6 ± 1.3
1h	2.58 ± 0.23	1.89 ± 0.29	4.23 ± 0.74	39.5 ± 2.1

Supplementary Table 2. The comparison between this manuscript and the prior published results

Ref. No.	ligand	metal salt	Young's modulus (MPa)	Maximum strain (mm mm^{-1})	Healing temperature (℃)	Healing time (h)	Healing efficiency (%)	toughness (MJ m^{-3})
this work	Hbimcp	$ZnCl_2$	43.68 ± 3.27	24	25 (R.T.)	24	98.9 ± 1.9	29.3
1	pdca	$FeCl_3$	0.54 ± 0.1	45 ± 0.2	25 (R.T.)	48	90.0±3.0	3.96[a]
2	NNN	$ZnCl_2$	0.12 ± 0.01	2.3 ± 0.26	25 (R.T.)	12	91.3 ± 2.2	0.15± 0.01
	Me-NNN	$ZnCl_2$	0.067 ± 0.012	4.56 ± 0.33	25 (R.T.)	0.5	94.7 ± 2.8	0.23±0.03
3	triazole	$CoCl_2$	1.12	5.7	120	24	47.3	3.17[a]
4	triazole	$FeCl_3$	0.46 ± 0.1	34	60	20	94.3	6.81[a]
5	bpy	$FeCl_2$	0.9± 0.2	1.25±0.20	25 (R.T.)	48	9[a]	0.52[a]
	bpy	$Fe(BF_4)_2$	1.0± 0.15	1.10 ± 0.16	25 (R.T.)	48	12[a]	0.18[a]
	bpy	$ZnCl_2$	1.2± 0.21	1.43±0.2	25 (R.T.)	48	21 ± 3	0.57[a]
	bpy	$Zn(ClO_4)_2$	1.2± 0.15	2.95 ± 0.17	25 (R.T.)	48	55 ± 21	1.98[a]
	bpy	$Zn(OTf)_2$	1.1± 0.2	3.1 ± 0.15	25 (R.T.)	48	76 ± 22	2.53[a]
6	bpy	$Eu(NO_3)_3$	1.2 ± 0.14	1.05± 0.2	25 (R.T.)	24	35[a]	0.62[a]
	bpy	$Eu(OTf)_3$	1.0± 0.26	3.00±0.1	25 (R.T.)	24	94[a]	0.54[a]
	bpy	$Tb(NO_3)_3$	1.2± 0.18	1.20±0.20	25 (R.T.)	24	32[a]	0.54[a]
	bpy	$Tb(OTf)_3$	1.0± 0.08	2.90±0.15	25 (R.T.)	24	96[a]	0.57[a]

Notes: [a] This data was estimated according to the data reported in the reference.

References: 1) Li C. H., *et al.* A highly stretchable autonomous self-healing elastomer. *Nat. Chem.* **8**, 618-624 (2016); 2) Wang D. P., Lai J. C., Lai H. Y., Mo S. R., Zeng K. Y., Li C. H., Zuo J. L. Distinct Mechanical and Self-Healing Properties in Two Polydimethylsiloxane Coordination Polymers with Fine-Tuned Bond Strength. *Inorg. Chem.* **57**, 3232−3242 (2018); 3) Jia X. Y., Mei J. F., Lai J. C., Li C. H., You X. Z. A self-healing PDMS polymer with solvatochromic properties. Chem. Commun. 51, 8928-8930 (2015); 4) Jia X. Y., Mei J. F., Lai J. C., Li C. H., You X. Z. A Highly Stretchable Polymer that Can Be Thermally Healed at Mild Temperature. *Macromol. Rapid Comm.* **37**, 952-956 (2016); 5) Rao Y.L., *et al.* Stretchable Self-Healing Polymeric Dielectrics Cross-Linked Through Metal-Ligand Coordination. *J. Am. Chem. Soc.* **138**, 6020-6027 (2016); 6) Rao Y. L., Feig V., Gu X., Wang G.J., Bao Z. The effects of counter anions on the dynamic mechanical response in polymer networks crosslinked by metal–ligand coordination. *J. Polym. Sci., Part A: Polym. Chem.* **55**, 3110-3116 (2017)

Supplementary Table 3. Crystallographic Data for complexes.

	$[Zn(Pr\text{-}Hbimcp)Cl_2]$	$[Zn_2(Pr\text{-}bimcp)_2Cl_2]$	$[Zn_2(Pr\text{-}bimcp)(Pr\text{-}Hbimcp)_2(CH_3O)](ClO_4)_2$
formula	$C_{14}H_{19}Cl_3N_2O_1Zn_1$	$C_{28}H_{36}Cl_4N_4O_2Zn_2$	$C_{43}H_{57}Cl_5N_6O_{12}Zn_2$
Mw	403.03	733.15	1154.91
crystal system	Triclinic	Trigonal	Monoclinic
space group	*P-1*	*R-3*	$P2_1/c$
a, Å	9.1694(8)	27.549(2)	14.661(3)
b, Å	12.4274(12)	27.549(2)	16.709(3)
c, Å	16.9159(16)	10.4297(17)	25.092(4)
α, °	103.905(2)	90.00	90.00
β, °	93.956(2)	90.00	117.951(8)
γ, °	103.467(2)	120.00	90.00
V, Å^3	1803.7 (3)	6855.3 (14)	5429.8(17)
Z	2	9	4
ρ_{calc}, g cm^{-3}	1.484	1.598	1.413
μ, mm^{-1}	1.805	1.959	1.189
GOF (F^2)	1.045	0.937	1.070
$R_1{}^a$,$wR_2{}^b$ (I>2σ(I))	0.0708, 0. 2071	0.0708, 0. 2071	0.0645, 0.1662
$R_1{}^a$,$wR_2{}^b$ (all data)	0.1027, 0. 2238	0.1027, 0. 2238	0.1524, 0.2177

$R_1{}^a = \Sigma||F_o| - |F_c||/\Sigma F_o|$, $wR_2{}^b = [\Sigma w(F_o{}^2 - F_c{}^2)^2/\Sigma w(F_o{}^2)^2]^{1/2}$

Supplementary Table 4. Selected bond lengths (Å) and angles (°) for complex [Zn(Pr-Hbimcp)Cl_2].

**Bond Distances (Å)**			
Zn(1)-N(1)	2.009(3)	Zn(1)-O(1)	1.969(3)
Zn(1)-Cl(1)	2.2248(11)	Zn(1)-C1(2)	2.2389(12)
**Bond Angles (°)**			
C7 N1 Zn1	123.0(3)	C8 N1 Zn1	119.2(3)
C4 O1 Zn1	126.7(2)	O1 Zn1 N1	9.71(12)
O1 Zn1 Cl1	108.81(10)	N1 Zn1 Cl1	118.51(10)
O1 Zn1 Cl2	110.14(10)	N1 Zn1 Cl2	108.75(10)
Cl1 Zn1 Cl2	114.80(4)		

Supplementary Table 5. Selected bond lengths (Å) and angles (°) for complex [Zn_2(Pr-bimcp)$_2Cl_2$].

**Bond Distances (Å)**			
Zn(1)-N(1)	2.057(6)	Zn(1)-N(2)	2.070(5)
Zn(1)-O(1)	2.052(4)	Zn(1)-O(2)	2.061(4)
Zn(1)-Cl(1)	2.226(3)		
**Bond Angles (°)**			
C11 N1 Zn1	126.1(5)	C13 N1 Zn1	120.6(8)
C12 N2 Zn1	125.4(5)	C16 N2 Zn1	120.0(15)
C1 O1 Zn1	128.6(4)	C1 O1 Zn1	127.9(4)
Zn1 O1 Zn1	103.52(18)	O1 Zn1 N1	145.4(2)
O1 Zn1 O1	76.48(18)	N1 Zn1 O1	86.1(2)
O1 Zn1 N2	85.8(2)	N1 Zn1 N2	92.2(2)
O1 Zn1 N2	145.0(2)	O1 Zn1 Cl1	109.09(15)
N1 Zn1 Cl1	104.8(2)	O1 Zn1 Cl1	109.49(15)
N2 Zn1 Cl1	104.7(2)		

Supplementary Table 6. Selected bond lengths (Å) and angles (°) for complex $[Zn_2$(Pr-bimcp)(Pr-Hbimcp)$_2(CH_3O)](ClO_4)_2$

**Bond Distances (Å)**			
Zn(1)-O(1)	2.072(4)	Zn(1)-O(2)	1.983(4)
Zn(1)-O(3)	2.069(4)	Zn(1)-N(1)	2.059(6)
Zn(1)-N(3)	2.063(5)	Zn(2)-O(1)	2.086(4)
Zn(2)-O(2)	1.970(4)	Zn(2)-O(4)	2.050(5)
Zn(2)-N(4)	2.094(6)	Zn(2)-N(5)	2.037(6)
**Bond Angles (°)**			
O3 Zn1 O1	170.49(19)	O2 Zn1 O1	75.81(17)
O2 Zn1 O3	96.04(18)	O2 Zn1 N3	135.6(2)
O2 Zn1 N1	105.5(2)	N3 Zn1 O1	85.25(19)
N3 Zn1 O3	97.62(2)	N1 Zn1 O1	97.6(2)
N1 Zn1 O3	89.2(2)	N1 Zn1 N3	116.7(2)
O1 Zn2 N4	82.22(19)	O2 Zn2 O1	75.77(17)
O2 Zn2 O4	93.72(19)	O2 Zn2 N4	145.2(2)
O2 Zn2 N5	109.8(2)	O4 Zn2 O1	159.02(19)
O4 Zn2 N4	97.7(2)	N5 Zn2 O1	109.6(2)
N5 Zn2 O4	90.9(2)	N5 Zn2 N4	102.8(2)
Zn1 O1 Zn2	99.96(17)		

Supplementary References

[S1] SAINT-Plus, version 6.02, Bruker Analytical X-ray System, Madison,WI (1999).

[S2] Sheldrick S.G.M., An Empirical Absorption Correction Program, Bruker Analytical X-ray Systems, Madison, WI (1996).

[S3] Shelxtl B. A., Madison W., GM Sheldrick, SHELX-97, *Acta Crystallogr. Sect. A: Fundam. Crystallogr.* **64**, 112–122 (2008).
