# Supplementary material for: Thermodynamically stable whilst kinetically labile coordination bonds lead to strong and tough self-healing polymers
Source: Nat Commun. 2019 Mar 11;10:1164. doi: 10.1038/s41467-019-09130-z (PMC6411951; doi:10.1038/s41467-019-09130-z)
Supplement: Supplementary file 3 — Description of Additional Supplementary Files [file 41467_2019_9130_MOESM3_ESM.pdf]

Description of Additional Supplementary Files

File Name: Supplementary Movie 1
Description: The comparison of composite sponge and blank sponge when deforming and recovering.
